# Genome-wide identification of lysin motif containing protein family genes in eight rosaceae species, and expression analysis in response to pathogenic fungus *Botryosphaeria dothidea* in Chinese white pear

**DOI:** 10.1186/s12864-020-07032-9

**Published:** 2020-09-07

**Authors:** Qiming Chen, Qionghou Li, Xin Qiao, Hao Yin, Shaoling Zhang

**Affiliations:** State Key Laboratory of Crop Genetics and Germplasm Enhancement, Centre of Pear Engineering Technology Research, Nanjing Agricultural University, Nanjing, China

**Keywords:** Chinese white pear, Lysin motif containing protein, Comparative analysis, Gene family, Evolution, Fungal pathogen resistance

## Abstract

**Background:**

Lysin motif-containing proteins (LYP), which act as pattern-recognition receptors, play central roles in growth, node formation, and responses to biotic stresses. The sequence of Chinese white pear genome (cv. ‘Dangshansuli’) along with the seven other species of Rosaceae has already been reported. Although, in these fruit crops, there is still a lack of clarity regarding the LYP family genes and their evolutionary history.

**Results:**

In the existing study, eight Rosaceae species i.e.*, Pyrus communis*, *Prunus persica*, *Fragaria vesca*, *Pyrus bretschneideri, Prunus avium, Prunus mume*, *Rubus occidentalis*, and *Malus × domestica* were evaluated. Here, we determined a total of 124 *LYP* genes from the underlined Rosaceae species. While eighteen of the genes were from Chinese white pear, named as *PbrLYPs*. According to the *LYPs* structural characteristics and their phylogenetic analysis, those genes were classified into eight groups (group LYK1, LYK2, LYK3, LYK4/5, LYM1/3, LYM2, NFP, and WAKL). Dispersed duplication and whole-genome duplication (WGD) were found to be the most contributing factors of *LYP* family expansion in the Rosaceae species. More than half of the duplicated *PbrLYP* gene pairs were dated back to the ancient WGD (~ 140 million years ago (MYA)), and *PbrLYP* genes have experienced long-term purifying selection. The transcriptomic results indicated that the *PbrLYP* genes expression was tissue-specific. Most *PbrLYP* genes showed differential expression in leaves under fungal pathogen infection with two of them located in the plasmalemma.

**Conclusion:**

A comprehensive analysis identified 124 *LYP* genes in eight Rosaceae species. Our findings have provided insights into the functions and characteristics of the Rosaceae *LYP* genes and a guide for the identification of other candidate *LYPs* for further genetic improvements for pathogen-resistance in higher plants.

## Background

In contrast to mammals, plants have no sophisticated mobile defender cells or a somatic adaptive immune system. Plants have been developed their survival strategies, depending on the innate immunity along with signals arising from the site of infection via pathogen co-evolution [1, 2]. Similar to other organisms, plants can recognize PAMPs via recruiting plasmalemma localized pattern-recognition receptors (PRRs) to initiate immune reactions, such as PAMP-triggered immunity (PTI) responses [3]. Upon the perception between ectodomain and corresponding ligand, the cytoplasmic kinase domain (KD) of PRRs could transmit the signal to downstream and activate defense responses, such as reactive oxygen species production (ROS), phytoalexins, accumulation of callose, as well the stimulation of MAPK (Mitogen-activated protein kinase) pathways and the pathogenesis-related (PR) proteins expression.

Plant PRRs could be divided in two clusters: Receptor-like kinases (RLKs), which contain an extracellular sensor domain, a transmembrane domain and an intracellular domain with homology to protein kinases, involved in signal transduction; Receptor-like proteins (RLPs), which are similar to RLKs but lack intracellular region [4]. RLK/Ps are firstly reported in animals, but the gene number is particularly expanded in plants [5]. As a plant specific PRR family, the functions of lysin motif (LysM) containing proteins (LYPs) in fungal and bacterial microbe perceptions have been well studied in rice and *Arabidopsis*. LYPs are common in land plants and may have evolved before land colonization and symbiosis with mycorrhiza as a signaling module [6, 7], and most of LYPs that have been characterized were related to the perception of *N*-acetyl glucosamine containing molecules and/or to be involved in plant-microbe interaction pathway including activating of defense responses and establishment of root endosymbioses. For example, the *Arabidopsis* genome encodes five LysM-RLKs, and three of them participate in chitin signaling with chitin affinity: AtCERK1 or LYK1, LYK4 and LYK5 [7–12]. AtCERK1 is essential for chitin signaling pathway in *Arabidopsis* by forming hetero-oligomeric complexes with LYK5 to initiate downstream PTI, and LYK4 is also involved in that pathway having functions partly redundant with LYK5. While OsCEBiP, the main chitin binding protein in rice, recruits OsCERK1 to activate the chitin-triggered immune responses [13–15]. In addition to activating innate immunity, LYPs in legumes are essential receptors for the perception of nodulation factors (NFs) released by rhizobia and the establishment of nitrogen fixing symbiosis [16–21].

Few LYPs have been previously reported in fruit trees including apple (*MdCERK1* and *MdCERK1–2*). *MdCERK1*, the ortholog of *AtCERK1*, has been shown to directly bind chitin and to be involved in transcriptional responses to pathogen infection of a soilborne pathogen *Rhizoctonia solani* [22]. *MdCERK1–2* is also involved in the anti-fungal defense responses as a PRR and significantly upregulated after *Botryosphaeria dothidea* infection [23]. However, for other therophyte and perennial species in the Rosaceae, members of the *LYP* gene family involved in fungal pathogen perception and their evolutionary history are poorly defined.

In this study, we identified the Rosaceae *LYP* genes at the genome-wide scale by employing bioinformatics and publicly available data, and analyzed part of their functions in pear. We annotated full-length *LYP* genes in pear and other Rosaceae species, investigated their subcellular localization, and analyzed their expression patterns in different pear tissue types. We investigated the expression of *PbrLYPs* in response to the infection by a fungal pathogen *Botryosphaeria dothidea*, and provided a relatively complete profile of the *LYP* gene family in the Rosaceae. The genetic structure, evolutionary analysis, and experimental data of *LYPs* provide potential candidate *LYPs* for the future genetic modifications of pathogen-resistance in Rosaceae fruit crops and other higher plants.

## Results

### Identification and classification of *LYP* genes in the Rosaceae

To identify the members of *LYP* gene family in the genus Rosaceae, HMM search was performed using both the HMM profile (PF01476) and a self-built HMM model against the whole-genome protein sequences of each species. A total of 141 *LYP* genes were identified from eight investigated Rosaceae species. After removing redundant and incomplete gene sequences, the longest transcript of the same gene was retained. Subsequently, the NCBI Batch CD-Search was used to further confirm the presence of a LysM domain (Table 1 and Fig. S3a). Finally, we identified 124 *LYP* genes in eight Rosaceae species, including 18 genes in Chinese white pear, 14 in European pear, 21 in apple, 15 in peach, 13 in strawberry, 16 in Mei (Japanese apricot), 14 in sweet cherry and 13 in black raspberry. The *PbrLYP* genes showed a random distribution on eight of the 17 chromosomes and three unanchored scaffolds (scaffolds681.0, scaffolds831.0, and scaffolds897.0) in pear (Supplementary Fig. S1).

**Table 1:** Classification of *LYP* genes in eight Rosaceae species

Group name		Chinese white pear	European pear	Apple	Strawberry	Peach	Mei	Sweet cherry	Black rasberry
(Number of genes)		Pbr	Pco	Md	Fv	Ppe	Pm	Pav	Ro
LYK1	LYK1a	*Pbr000107.1*	*PCP017929.1*	*MD05G1351500*	*FvH4_3g01490.1*	*ppa017142m*	*Pm009697*	*Pav_sc0000824.1_g060.1.mk*	*Ro03_G13508*
(22)	LYK1b1	*Pbr005151.1*	*PCP003883.1*	*MD09G1111800*	*FvH4_6g42640.1*	*ppa003023m*	*Pm015128*	*Pav_sc0001077.1_g520.1.mk*	*Ro06_G06708*
	LYK1b2	*Pbr005152.1*	*PCP008886.1*	*MD17G1102100*			*Pm015129*		*Ro06_G17308*
	LYK1b3	*Pbr021830.1*							
LYK2	LYK2	*Pbr014439.4*	*PCP023229.1*	*MD17G1014100*	*FvH4_6g53170.1*	*ppa024632m*	*Pm016182*		*Ro06_G03822*
(7)									
LYK3	LYK3a1	*Pbr019107.1*	*PCP003907.1*	*MD09G1202500*	*FvH4_6g32430.1*	*ppa002771m*	*Pm013417*	*Pav_sc0000759.1_g060.1.mk*	*Ro06_G20150*
(21)	LYK3a2	*Pbr034737.1*	*PCP003981.1*	*MD17G1183700*				*Pav_sc0002893.1_g120.1.mk*	
	LYK3b1	*Pbr036151.1*	*PCP000376.1*	*MD16G1098600*		*ppa019853m*	*Pm006733*	*Pav_sc0006018.1_g500.1.mk*	*Ro04_G02801*
	LYK3b2		*PCP002145.1*	*MD06G1169200*					
LYK4/5	LYK4/5a1	*Pbr022856.1*		*MD02G1156000*	*FvH4_1g14190.1*	*ppa002539m*	*Pm022870*		*Ro05_G22711*
(24)	LYK4/5a2				*FvH4_5g26710.1*	*ppa015910m*	*Pm026556*		
	LYK4/5a3				*FvH4_5g26740.1*				
	LYK4/5b	*Pbr022855.1*			*FvH4_1g14260.1*	*ppa016660m*	*Pm026555*	*Pav_sc0000348.1_g1000.1.mk*	*Ro01_G00968*
	LYK4/5c1		*PCP028343.1*	*MD00G1205900*	*FvH4_3g31710.1*	*ppa027141m*	*Pm001054*	*Pav_sc0000480.1_g310.1.mk*	*Ro03_G07424*
	LYK4/5c2			*MD11G1140300*					
NFP	NFP1	*Pbr014715.1*		*MD02G1156500*	*FvH4_1g14270.1*	*ppa002872m*	*Pm009175*	*Pav_sc0000998.1_g140.1.mk*	*Ro01_G00967*
(12)	NFP2	*Pbr022854.1*		*MD13G1203700*		*ppa002949m*	*Pm026553*	*Pav_sc0000348.1_g980.1.mk*	
WAKL	WAKL1	*Pbr035317.1*	*PCP000222.1*	*MD04G1238700*		*ppa002979m*	*Pm003663*	*Pav_sc0000744.1_g060.1.mk*	
(11)	WAKL2		*PCP038026.1*	*MD04G1238900*				*Pav_sc0004456.1_g300.1.br*	
	WAKL3			*MD04G1239400*					
	WAKL4			*MD04G1239000*					
LYM1/3	LYM1/3–1	*Pbr002167.1*	*PCP020213.1*	*MD06G1215700*	*FvH4_2g26750.1*	*ppa006147m*	*Pm004817*	*Pav_sc0000195.1_g1050.1.mk*	*Ro02_G01432*
(18)	LYM1/3–2	*Pbr020280.1*	*PCP035131.1*	*MD08G1165500*	*FvH4_5g12220.1*	*ppa017855m*	*Pm025193*	*Pav_sc0000383.1_g420.1.mk*	*Ro05_G16294*
	LYM1/3–3	*Pbr040557.1*		*MD15G1351200*					
LYM2	LYM2–1	*Pbr016347.1*	*PCP000950.1*	*MD00G1083800*	*FvH4_2g16390.1*	*ppa008092m*	*Pm020791*	*Pav_sc0000377.1_g320.1.mk*	*Ro02_G18370*
(9)	LYM2–2	*Pbr039238.2*							

**Fig. 1:**
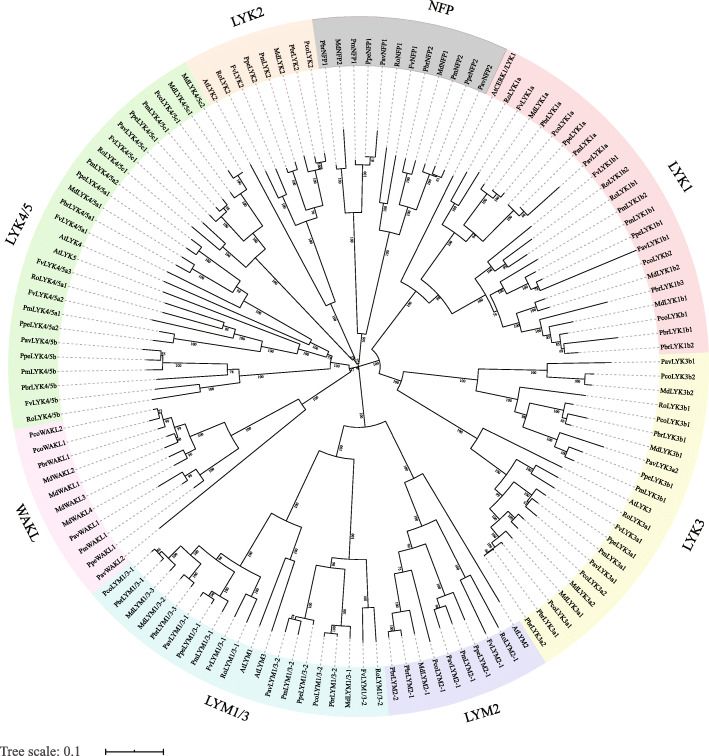
Phylogenetic analysis of *LYP* genes in Rosaceae and *Arabidopsis*. The tree was formed via MEGA 7.0 with the NJ method and 1000 bootstraping replicates. The proteins were assembled into eight groups. Different background colors indicate the different groups of the LYP proteins

Phylogenetic analyses of the LYP protein sequences were performed in order to classify the *LYP* genes and investigate their evolutionary relationships. The phylogenetic tree showed that the *LYP* genes are separated into eight well-supported clades. According to the name of the best hit gene in *Arabidopsis*, these subfamilies were named LYK1–3, LYK4/5, LYM1/3, LYM2, NFP (Nod factor perception protein), and WAKL (Wall associated kinase-like) (Fig. 1). The subfamily classification and corresponding names of *LYPs* are shown in Table 1. Although, the best local BLASTP hit gene of most LYPs in NFP clade were AtLYK4 or AtLYK5, their best NCBI BLASTP results were categorized as NFP (data not shown). Notably, the numbers of genes in the LYK1, LYK3 and LYK4/5 clades were more than that of others, suggesting these three subgroups may have undergone the subfamily specific expansion.

**Fig. 2:**
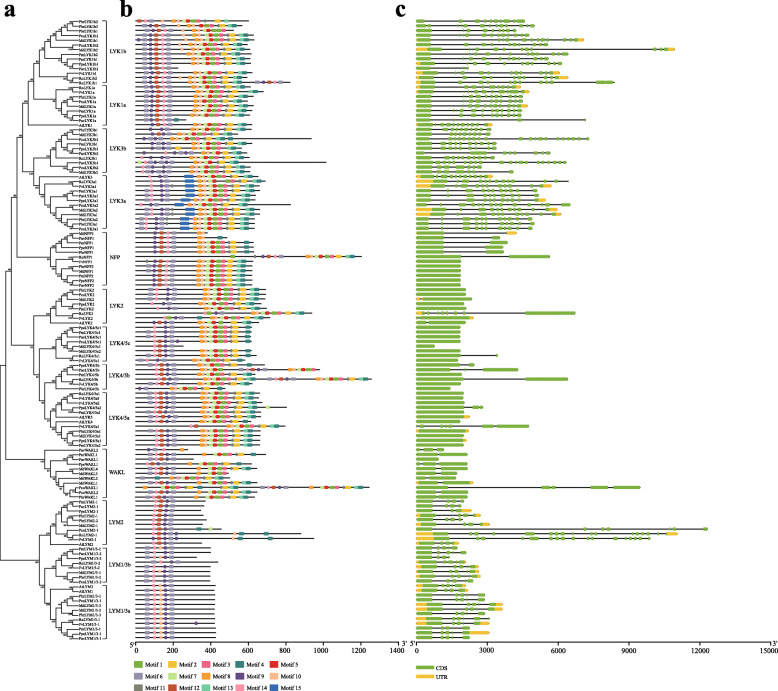
Structural and motif analysis of *LYP* genes. **a** Subgroup classification. Neighbor-joining phylogenetic tree was generated among 132 *LYP* genes with MEGA7. The subgroup names were labeled accordingly. **b** Motif analysis. Fifteen distinct motifs were determined with MEME suite and the representation of each motif was carried out with a different color. **c** Gene structural analysis. The exon sizes are comparable to their sequence length

To explore the structural diversity of Rosaceae *LYP* genes, an exon distribution analysis was performed (Fig. 2). The results showed that these Rosaceae LYP subgroups displayed different exon abundance and the numbers of exons in each gene in the same subgroup were similar, supporting the phylogenetic classification of the LYP genes (Fig. 2a, c). However, among the Rosaceae, the number of exons in subgroups LYK1 and LYK3 was much higher compared to others, about 11 on average. These results were consistent with *Arabidopsis*. Exon number were relatively conserved in subgroups LYM1/3 and LYM2, at about 4 to 5 (Table 2 and Supplementary Table S1). These results were well consistent with previous reports about the conserved exon number of different LYP types. Type I LYP genes contained was up to 10 exons (group LYK1 and LYK3), type II LYP contained approximately 5 (group LYM1/3 and LYM2), and of type III contained approximately 2 (group LYK2, LYK4/5, NFP and WAKL) [10, 24–26]. The conserved and specific exon numbers of certain LYP type may have been due to similar replication events, implying that the different LYP type genes originated through the evolutionary path separate from genes in other types.

**Table 2:** Characteristics of the LYP proteins

Gene name	Coding sequence length (bp)	Pprotein length (aa)	MW	PI	GRAVY	Extron number	PDB domain 4EBZ	CDD domain LysM	Best hit gene in *Arabidopsis*	Corresponding gene name in *Arabidopsis*
*AtCERK1/LYK1*	1854	618aa	67.32 kDa	6.79	−0.012*	12	D	D	AT3G21630.1	LYK1
*AtLYK2*	1965	655aa	73.17 kDa	6.18	−0.291*	2	D	D	AT3G01840.1	LYK2
*AtLYK3*	1956	652aa	71.45 kDa	6.28	−0.004*	11	D	D	AT1G51940.1	LYK3
*AtLYK4*	1839	613aa	66.63 kDa	5.13	−0.057*	1	D	D	AT2G23770.1	LYK4
*AtLYK5*	1995	665aa	72.58 kDa	5.84	−0.117*	1	D	D	AT2G33580.1	LYK5
*AtLYM1*	1251	417aa	43.49 kDa	4.65	0.293	5	D	D	AT1G21880.2	LYM1
*AtLYM2*	1053	351aa	37.75 kDa	6.23	0.04	4	D	D	AT2G17120.1	LYM2
*AtLYM3*	1272	424aa	44.16 kDa	4.77	0.243	5	D	D	AT1G77630.1	LYM3
*FvLYK1a*	2037	679aa	75.29 kDa	6.26	−0.054*	10	D	D	AT3G21630.1	LYK1
*FvLYK1b1*	1860	620aa	68.52 kDa	4.94	−0.2*	12	D		AT3G21630.1	LYK1
*FvLYK2*	2142	714aa	78.52 kDa	6.84	−0.03*	2	D	D	AT3G01840.1	LYK2
*FvLYK3a1*	1974	658aa	72.71 kDa	6.51	−0.047*	11	D	D	AT1G51940.1	LYK3
*FvLYK4/5a1*	2385	795aa	87.54 kDa	6.82	0.021	7	D		AT2G23770.1	LYK4
*FvLYK4/5a2*	2016	672aa	73.26 kDa	6.23	−0.113*	1	D	D	AT2G33580.1	LYK5
*FvLYK4/5a3*	1965	655aa	70.90 kDa	6.14	−0.138*	1	D	D	AT2G33580.1	LYK5
*FvLYK4/5b*	1860	620aa	69.09 kDa	6.72	0.04	1	D	D	AT2G23770.1	LYK4
*FvLYK4/5c1*	1743	581aa	64.21 kDa	6.39	0.041	1	D	D	AT2G23770.1	LYK4
*FvLYM1/3–1*	1257	419aa	43.65 kDa	4.98	0.268	5	D	D	AT1G21880.2	LYM1
*FvLYM1/3–2*	1191	397aa	42.60 kDa	6.32	0.181	5	D	D	AT1G21880.2	LYM1
*FvLYM2–1*	2847	949aa	101.63 kDa	7.62*	0.323	17	D	D	AT4G38380.1	MATE efflux family protein
*FvNFP1*	1872	624aa	68.95 kDa	8.05*	0.105	1	D	D	AT2G33580.1	LYK5
*MdLYK1a*	1878	626aa	68.98 kDa	6.37	−0.112*	12	D		AT3G21630.1	LYK1
*MdLYK1b1*	1890	630aa	69.54 kDa	5.03	−0.09*	12	D		AT3G21630.1	LYK1
*MdLYK1b2*	1824	608aa	66.72 kDa	5.38	−0.12*	12	D	D	AT3G21630.1	LYK1
*MdLYK2*	2067	689aa	75.24 kDa	6.92	−0.069*	1	D	D	AT3G01840.1	LYK2
*MdLYK3a1*	1980	660aa	72.94 kDa	7.13*	−0.066*	11	D	D	AT1G51940.1	LYK3
*MdLYK3a2*	1980	660aa	72.65 kDa	6.27	−0.056*	11	D	D	AT1G51940.1	LYK3
*MdLYK3b1*	1629	543aa	60.77 kDa	5.73	−0.109*	9	D	D	AT1G51940.1	LYK3
*MdLYK3b2*	1824	608aa	68.76 kDa	6.18	−0.267*	7	D	D	AT1G51940.1	LYK3
*MdLYK4/5a1*	1986	662aa	72.19 kDa	4.87	−0.074*	1	D	D	AT2G23770.1	LYK4
*MdLYK4/5c1*	756	252aa	27.80 kDa	4.43	0.04	1	D	D	AT2G23770.1	LYK4
*MdLYK4/5c2*	1845	615aa	68.29 kDa	6.71	0.002	1	D	D	AT2G23770.1	LYK4
*MdLYM1/3–1*	1185	395aa	42.24 kDa	6.37	0.255	5	D	D	AT1G21880.2	LYM1
*MdLYM1/3–2*	1254	418aa	43.49 kDa	4.7	0.368	5	D	D	AT1G21880.2	LYM1
*MdLYM1/3–3*	1260	420aa	43.85 kDa	5.99	0.374	5	D	D	AT1G21880.2	LYM1
*MdLYM2–1*	1068	356aa	38.31 kDa	7.25*	0.09	4	D	D	AT2G17120.1	LYM2
*MdNFP1*	1860	620aa	69.00 kDa	6.98	0.017	1	D	D	AT2G33580.1	LYK5
*MdNFP2*	1143	381aa	41.79 kDa	8.29*	−0.006*	2	D	D	AT2G23770.1	LYK4
*MdWAKL1*	1938	646aa	72.01 kDa	5.94	−0.121*	2	D	D	AT1G16120.1	WAKL1
*MdWAKL2*	1500	500aa	56.17 kDa	6.83	−0.207*	2	D	D	AT1G16130.1	WAKL2
*MdWAKL3*	1479	493aa	54.68 kDa	7.8*	0.006	2	D	D	AT1G16120.1	WAKL1
*MdWAKL4*	1935	645aa	71.87 kDa	6.32	−0.103*	2	D	D	AT1G16150.1	WAKL4
*PavLYK1a*	801	267aa	30.13 kDa	7.74*	−0.049*	4	D		AT3G21630.1	LYK1
*PavLYK1b1*	675	225aa	25.03 kDa	6.73	−0.144*	3	D	D	AT3G21630.1	LYK1
*PavLYK3a1*	1914	638aa	70.56 kDa	6.56	−0.003*	10	D	D	AT1G51940.1	LYK3
*PavLYK3a2*	1776	592aa	66.34 kDa	4.99	−0.107*	8	D	D	AT1G51940.1	LYK3
*PavLYK3b1*	3042	1014aa	115.03 kDa	6.74	−0.214*	20	D	D	AT1G60780.1	HAPLESS 13
*PavLYK4/5b*	2940	980aa	108.71 kDa	7.2*	−0.106*	3	D	D	AT2G23770.1	LYK4
*PavLYK4/5c1*	1836	612aa	68.08 kDa	6.1	0.011	1	D	D	AT2G23770.1	LYK4
*PavLYM1/3–1*	1275	425aa	44.24 kDa	6.32	0.324	5	D	D	AT1G21880.2	LYM1
*PavLYM1/3–2*	1194	398aa	42.74 kDa	6.02	0.095	5	D	D	AT1G21880.2	LYM1
*PavLYM2–1*	1089	363aa	38.78 kDa	7.7*	0.108	4	D	D	AT2G17120.1	LYM2
*PavNFP1*	1455	485aa	53.72 kDa	8.02*	−0.094*	2	D	D	AT2G23770.1	LYK4
*PavNFP2*	1857	619aa	68.76 kDa	8.13*	0.012	1	D	D	AT2G33580.1	LYK5
*PavWAKL1*	921	307aa	33.36 kDa	7.97*	0.097	1	D	D	AT2G33580.1	LYK5
*PavWAKL2*	828	276aa	30.46 kDa	8.75*	−0.103*	3	D	D	AT2G23770.1	LYK4
*PbrLYK1a*	1878	626aa	68.85 kDa	6.31	−0.088*	12	D		AT3G21630.1	LYK1
*PbrLYK1b1*	1563	521aa	57.39 kDa	7.51*	−0.188*	9	D	D	AT3G21630.1	LYK1
*PbrLYK1b2*	1800	600aa	65.81 kDa	5.97	−0.137*	10	D	D	AT3G21630.1	LYK1
*PbrLYK1b3*	1698	566aa	61.85 kDa	8.5*	−0.199*	13	D		AT3G21630.1	LYK1
*PbrLYK2*	2076	692aa	75.39 kDa	6.74	−0.084*	1	D	D	AT3G01840.1	LYK2
*PbrLYK3a1*	1896	632aa	69.70 kDa	6.73	−0.106*	11	D	D	AT1G51940.1	LYK3
*PbrLYK3a2*	1896	632aa	69.64 kDa	6.85	−0.108*	11	D	D	AT1G51940.1	LYK3
*PbrLYK3b1*	1845	615aa	68.86 kDa	6.43	−0.12*	10	D	D	AT1G51940.1	LYK3
*PbrLYK4/5a1*	1989	663aa	72.36 kDa	5.07	−0.057*	1	D	D	AT2G23770.1	LYK4
*PbrLYK4/5b*	1428	476aa	52.71 kDa	7.34*	−0.05*	1	D		AT2G23770.1	LYK4
*PbrLYM1/3–1*	1263	421aa	43.73 kDa	5.99	0.343	5	D	D	AT1G21880.2	LYM1
*PbrLYM1/3–2*	1188	396aa	42.09 kDa	6.48	0.222	5	D	D	AT1G21880.2	LYM1
*PbrLYM1/3–3*	1254	418aa	43.48 kDa	5.53	0.361	5	D	D	AT1G21880.2	LYM1
*PbrLYM2–1*	1077	359aa	38.51 kDa	7.29*	0.149	4	D	D	AT2G17120.1	LYM2
*PbrLYM2–2*	1134	378aa	40.55 kDa	8.24*	0.165	4	D	D	AT2G17120.1	LYM2
*PbrNFP1*	1887	629aa	69.18 kDa	6.51	−0.066*	2	D	D	AT2G23770.1	LYK4
*PbrNFP2*	1860	620aa	69.04 kDa	8.3*	0.011	1	D	D	AT2G33580.1	LYK5
*PbrWAKL1*	1938	646aa	72.04 kDa	5.97	−0.148*	2	D	D	AT1G16130.1	WAKL2
*PcoLYK1a*	1785	595aa	65.41 kDa	5.93	−0.09*	11	D		AT3G21630.1	LYK1
*PcoLYK2*	2076	692aa	75.44 kDa	6.81	−0.088*	1	D	D	AT3G01840.1	LYK2
*PcoLYK3a1*	1893	631aa	69.78 kDa	6.73	−0.122*	11	D	D	AT1G51940.1	LYK3
*PcoLYK3a2*	2472	824aa	90.91 kDa	6.64	−0.179*	14	D	D	AT1G51940.1	LYK3
*PcoLYK3b1*	2805	935aa	106.20 kDa	5.58	−0.211*	20	D	D	AT1G60780.1	HAPLESS 13
*PcoLYK3b2*	1830	610aa	68.88 kDa	5.85	−0.242*	7	D	D	AT1G51940.1	LYK3
*PcoLYK4/5c1*	1845	615aa	68.30 kDa	6.5	0.015	1	D	D	AT2G23770.1	LYK4
*PcoLYKb1*	1881	627aa	68.55 kDa	5.4	−0.124*	12	D	D	AT3G21630.1	LYK1
*PcoLYKb2*	1782	594aa	65.09 kDa	5.51	−0.111*	10	D	D	AT3G21630.1	LYK1
*PcoLYM1/3–1*	1260	420aa	43.74 kDa	6.32	0.335	5	D	D	AT1G21880.2	LYM1
*PcoLYM1/3–2*	1185	395aa	42.11 kDa	6.48	0.199	5	D	D	AT1G21880.2	LYM1
*PcoLYM2–1*	1368	456aa	48.61 kDa	4.67	0.295	7	D	D	AT2G17120.1	LYM2
*PcoWAKL1*	3732	1244aa	138.78 kDa	5.9	−0.202*	5	D	D	AT1G16130.1	WAKL2
*PcoWAKL2*	1938	646aa	72.10 kDa	6.07	−0.164*	2	D	D	AT1G16130.1	WAKL2
*PmLYK1a*	1857	619aa	68.14 kDa	6.37	−0.009*	12	D		AT3G21630.1	LYK1
*PmLYK1b1*	1827	609aa	66.66 kDa	6.43	−0.106*	12	D	D	AT3G21630.1	LYK1
*PmLYK1b2*	1845	615aa	67.51 kDa	5.38	−0.085*	12	D	D	AT3G21630.1	LYK1
*PmLYK2*	2088	696aa	76.31 kDa	7.03*	−0.086*	1	D	D	AT3G01840.1	LYK2
*PmLYK3a1*	1980	660aa	72.77 kDa	6.31	−0.052*	11	D	D	AT1G51940.1	LYK3
*PmLYK3b1*	1854	618aa	69.19 kDa	5.9	−0.092*	10	D	D	AT1G51940.1	LYK3
*PmLYK4/5a1*	1995	665aa	72.37 kDa	6.91	−0.076*	1	D	D	AT2G33580.1	LYK5
*PmLYK4/5a2*	1980	660aa	72.78 kDa	5.24	−0.13*	1	D	D	AT2G23770.1	LYK4
*PmLYK4/5b*	1908	636aa	70.84 kDa	5.62	−0.021*	1	D		AT2G23770.1	LYK4
*PmLYK4/5c1*	1845	615aa	68.61 kDa	6.63	0.028	1	D	D	AT2G23770.1	LYK4
*PmLYM1/3–1*	1275	425aa	44.56 kDa	5.98	0.309	5	D	D	AT1G21880.2	LYM1
*PmLYM1/3–2*	1194	398aa	42.71 kDa	5.82	0.124	5	D	D	AT1G21880.2	LYM1
*PmLYM2–1*	1110	370aa	39.57 kDa	6.47	0.131	4	D	D	AT2G17120.1	LYM2
*PmNFP1*	1878	626aa	69.89 kDa	6.92	−0.098*	2	D	D	AT2G23770.1	LYK4
*PmNFP2*	1854	618aa	68.99 kDa	8.19*	−0.007*	1	D	D	AT2G33580.1	LYK5
*PmWAKL1*	2076	692aa	77.10 kDa	7.28*	−0.133*	2	D	D	AT1G16130.1	WAKL2
*PpeLYK1a*	1821	607aa	66.68 kDa	6.27	0.018	10	D		AT3G21630.1	LYK1
*PpeLYK1b1*	1836	612aa	66.63 kDa	6.36	−0.135*	12	D	D	AT3G21630.1	LYK1
*PpeLYK2*	2004	668aa	73.24 kDa	6.77	−0.174*	1	D	D	AT3G01840.1	LYK2
*PpeLYK3a1*	1908	636aa	70.35 kDa	6.67	−0.021*	10	D	D	AT1G51940.1	LYK3
*PpeLYK3b1*	1689	563aa	62.86 kDa	6.04	−0.109*	9	D	D	AT1G51940.1	LYK3
*PpeLYK4/5a1*	1983	661aa	72.33 kDa	4.98	−0.125*	1	D	D	AT2G23770.1	LYK4
*PpeLYK4/5a2*	2409	803aa	87.75 kDa	7.1*	−0.091*	3	D	D	AT2G33580.1	LYK5
*PpeLYK4/5b*	2058	686aa	76.08 kDa	5.71	−0.062*	2	D	D	AT2G33580.1	LYK5
*PpeLYK4/5c1*	1848	616aa	68.58 kDa	6.9	0.014	1	D	D	AT2G23770.1	LYK4
*PpeLYM1/3–1*	1278	426aa	44.26 kDa	5.98	0.324	5	D	D	AT1G21880.2	LYM1
*PpeLYM1/3–2*	972	324aa	34.79 kDa	6.62	0.171	4	D	D	AT1G21880.1	LYM1
*PpeLYM2–1*	1038	346aa	36.84 kDa	7.13*	0.125	3	D	D	AT2G17120.1	LYM2
*PpeNFP1*	1881	627aa	69.66 kDa	7.26*	−0.081*	2	D	D	AT2G23770.1	LYK4
*PpeNFP2*	1860	620aa	68.93 kDa	8.35*	−0.009*	1	D	D	AT2G33580.1	LYK5
*PpeWAKL1*	1848	616aa	68.83 kDa	7.02*	−0.19*	3	D	D	AT1G16130.1	WAKL2
*RoLYK1a*	1833	611aa	67.26 kDa	6.13	0.013	9	D	D	AT3G21630.1	LYK1
*RoLYK1b1*	2466	822aa	90.99 kDa	5.32	−0.044*	13	D	D	AT3G21630.1	LYK1
*RoLYK1b2*	1779	593aa	65.27 kDa	5.96	−0.027*	10	D	D	AT3G21630.1	LYK1
*RoLYK2*	2814	938aa	102.59 kDa	6.65	−0.03*	8	D	D	AT3G01840.1	LYK2
*RoLYK3a1*	2070	690aa	76.39 kDa	6.5	−0.029*	13	D	D	AT1G51940.1	LYK3
*RoLYK3b1*	1815	605aa	67.14 kDa	5.94	0.012	10	D	D	AT1G51940.1	LYK3
*RoLYK4/5a1*	1980	660aa	71.61 kDa	6.28	−0.099*	1	D	D	AT2G33580.1	LYK5
*RoLYK4/5b*	3765	1255aa	139.28 kDa	7.69*	−0.105*	2	D	D	AT2G23770.1	LYK4
*RoLYK4/5c1*	1926	642aa	71.38 kDa	6.76	−0.004*	2	D	D	AT2G23770.1	LYK4
*RoLYM1/3–1*	1260	420aa	43.57 kDa	4.67	0.262	6	D	D	AT1G21880.2	LYM1
*RoLYM1/3–2*	1311	437aa	47.21 kDa	7.97*	0.03	5	D	D	AT1G21880.2	LYM1
*RoLYM2–1*	2637	879aa	95.20 kDa	8.24*	0.244	17	D	D	AT4G38380.1	MATE efflux family protein
*RoNFP1*	3606	1202aa	135.40 kDa	8.21*	−0.118*	2	D	D	AT5G66631.1	Tetratricopeptide repeat like superfamily protein

The basic information and characteristics of LYP proteins and encoding genes were shown

MW: Protein Molecular Weight. PI: Isoelectric point. GRAVY: Grand average of hydropathy. 4EBZ: Crystal structure of the LysMs in the ectodomain of AtCERK1 in PDB databases (https://www.rcsb.org/). CDD domain LysM: The domain of LysM or LysM superfamily in CDD v3.18. Best hit gene in *Arabidopsis*: The gene ID with the highest score in BLASTP result

The symbol * indicate the values more than 7 or less than 0 and the letter D indicate the corresponding domain could be detected

### Features of the LYPs in the Rosaceae

The characteristics of the LYPs and their coding genes are shown in Table 2. The lengths of the LYPs protein sequences ranged from 225 to 1255 amino acids and the molecular weights were 25.03 to 139.28 kD. Protein isoelectric points (PI) ranged from 4.43 to 8.75, with the majority lower than 7 (Table 2). The highest number of exons in pear *LYPs* was found in the LYK1 and LYK3 subgroups. A similar trend was also observed in the other five Rosaceae species (European pear, apple, peach, sweet cherry and Mei) and *Arabidopsis* (Table 2), confirming that the genes in LYK1 and LYK3 groups have undergone specific evolutionary events as type I LYPs. However, the highest exon numbers in strawberry and black raspberry were detected in the LYM2 subgroup, suggesting that these species may have experienced an unknown evolutionary process or some specific selection forces. The grand average of hydropathy (GRAVY) for most LYK proteins in pear was positive, while that of LYMs was negative. The GRAVY of NFP and WAKL subgroups was random. A similar trend for all subgroups was also observed in the other Rosaceae species. These results indicated that similar to *Arabidopsis*, most of the LYK proteins are hydrophobic and all LYM proteins are hydrophilic in the *LYP* gene family (Table 2).

### Synteny analysis of LYPs

**Fig. 3:**
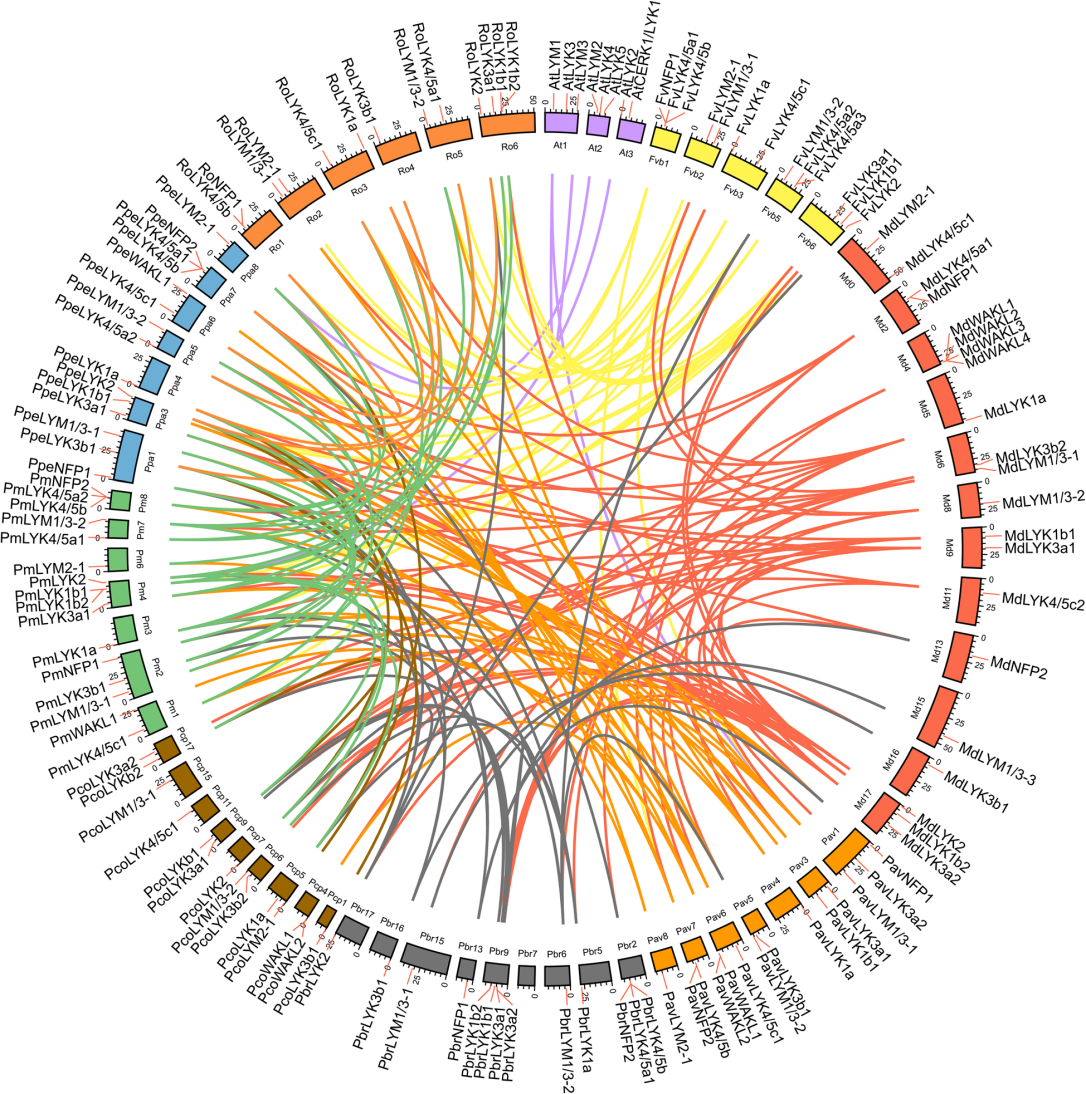
Distribution and collinearity of the *LYP* genes. Red lines along the circumference of the circle mark the gene positions. The lines in different colors inside the circle indicate collinearity relationships among the genes from *Arabidopsis* and eight Rosaceae species

**Fig. 4:**
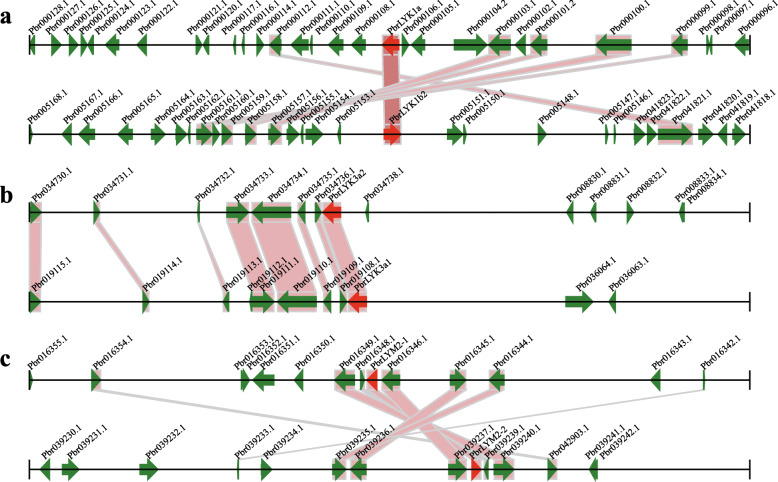
Segmental duplications between *PbrLYPs*. This figure depicts a stretch of nucleotides, comprising 100 kb on each side flanking the *LYP* genes. Pairs of homologous gene are linked with bands. The black horizontal line showed the chromosome segment, and the green broad line containing arrowhead indicates the gene along with its transcriptional orientation. The text beside the gene is the gene ID. The *LYP* genes are indicated in red, and other genes indicated in green. **a** *PbrLYK1a-PbrLYK1b2*, **b** *PbrLYK3a1-PbrLYK3a2*, **c** *PbrLYM2–1-PbrLYM2–2*

The gene duplication events, such as tandem duplication, the whole genome duplication (WGD)/segmental duplication, and transposition events are the contributing factors in gene family development that impact the protein-coding gene family’s evolution [27]. By MCScanX package, we detected the events duplication related to the LYP gene family, and assigned each of *LYP* genes to one of the five various types of duplication: WGD/segmental, singleton, proximal, dispersed, or tandem. In *Arabidopsis*, only two *LYP* genes duplicated during the WGD/segmental event, while the others originated from a dispersed duplication. Unlike in *Arabidopsis*, the five duplication types were all detected in the Rosaceae driving the expansion of the *LYP* genes (Table 3 and Supplementary Table S2). WGD occurred in all the Rosaceae species studied, with 38.9% of *LYP* genes in Chinese white pear and 57.2% in apple retained and duplicated from WGD/segmental events. However, the percentage of genes retained following dispersed duplication in peach (53.3%), strawberry (46.2%), Mei (56.3%), sweet cherry (57.1%), and black raspberry (69.2%) was higher than that in apple (19%). Peach, strawberry, Mei, sweet cherry, and black raspberry did experience a WGD from the time of their divergence from pear and apple. Hence, these species may have experienced more genome rearrangements and gene losses during the long-term evolution in the absence of WGD, resulting in the larger ratios of dispersed genes. Although pear and apple have undergone the same recent WGD event, Chinese white pear and European pear showed a higher percentage of dispersed *LYP* genes (38.9 and 50%, respectively) compared to apple. This may be due to the differences in the ratio of self-incompatibility and the domestication process between pear and apple. However, proximal duplication events of *LYP* genes were only detected in apple (14.3%), strawberry (23.2%), peach (6.7%), Mei (6.2%), and sweet cherry (14.3%) as depicted in Table 3. The obtained data suggested that WGD and dispersed gene duplication have an effective contribution to the development of *LYP* gene family, belong to Rosaceae. To reveal the *LYP* genes (belong to Rosaceae) evolutionary routes that made them the most diverse, here, we evaluated both intra- and intergenomic synteny analyses to identify conservation chromosome blocks within Chinese white pear and among eight Rosaceae species and *Arabidopsis*. The landscape of inter-species orthologous *LYP* gene pairs among Rosaceae species and *Arabidopsis* presented in Fig. 3 and their chromosomal distribution was random. In the Chinese white pear genome, 7 conserved syntenic blocks containing *PbrLYPs* were detected, including most of WGD/segmental type *LYP* gene pairs (*PbrLYK1a-PbrLYK1b2*, *PbrLYM2–1-PbrLYM2–2,* and *PbrLYK3a1-PbrLYK3a2*) (Fig. 4). The timing of the WGD/segmental duplication events could be estimated by the Ks value (synonymous substitutions per site) [28]. Based on previous reports, the Ks values, show that the genome of apple and pear have undergone two genome-wide duplication events: the ancient WGD from γ triplication (Ks ~ 1.6) and a recent WGD (Ks ~ 0.2) [29] in the apple genome, as well the ancient WGD (Ks ~ 1.5–1.8) that took place ~ 140 MYA [30] and the recent WGD (Ks ~ 0.15–0.3) occurred at 30–45 MYA [31] in pear. Hence, Ks values were used to estimate the time for the gene duplication events among the *PbLYP* gene family members. The Ks values suggest that most *PbrLYP* genes were duplicated from around the time of the ancient WGD event, while some originated from the recent WGD (Table 4). The Ka/Ks ratio represents the selection intensity and direction. The Ka/Ks value of one showed neutral evolution, positive selection when the Ka/Ks value is greater than one, and purifying selection when the Ka/Ks value is lower than one [32]. Our results showed all Ka/Ks ratios of the *PbrLYP* gene pairs were lower than one, demonstrating, *PbrLYPs* primarily evolved under purifying selection (Table 4).

**Table 3:** Numbers of *LYP* genes from different origins in *Arabidopsis* and Roseceae genomes

Species	No. of total LYP genes	No. of LYP genes from different origins (percentage)				
		Singleton	Dispersed	Proximal	Tandem	WGD/segmental
Arabidopsis	8	0 (0)	6 (75)	0 (0)	0 (0)	2 (25)
Chinese white pear	18	0 (0)	7 (39)	0 (0)	4 (22)	7 (39)
European pear	14	0 (0)	7 (50)	0 (0)	2 (14)	5 (36)
Apple	21	0 (0)	4 (19)	3 (14)	2 (10)	12 (57)
Strawberry	13	0 (0)	6 (46)	3 (23)	2 (15)	2 (15)
Peach	15	0 (0)	8 (53)	1 (7)	4 (27)	2 (13)
Mei	16	0 (0)	9 (56)	1 (6)	4 (25)	2 (13)
Sweet cherry	14	0 (0)	8 (57)	2 (14)	2 (14)	2 (14)
Black rasberry	13	0 (0)	9 (69)	0 (0)	2 (15)	2 (15)

Note: The table shows the total numbers of LYP genes and the numbers of genes from each kind of duplication events in Arabidopsis and eight Rosaceae species

**Table 4:** The duplicate mode and estimation of the absolute date for large-scale duplication events in Chines white pear

	Duplicated pair	Duplicated mode	Number of conserved flanking protein-coding genes	Mean Ka	Mean Ks	Date (million years ago)	Ka/Ks
LYK1	*PbrLYK1a — PbrLYK1b2*	WGD/segmental	6	0.322	2.047	684.62	0.16
	*PbrLYK1a — PbrLYK1b3*	Dispersed	\	0.356	2.093	700.28	0.17
	*PbrLYK1b1 — PbrLYK1b3*	Dispersed	\	0.111	0.183	61.35	0.61
	*PbrLYK1b2 — PbrLYK1b1*	Tandem	\	0.090	0.173	57.72	0.52
	*PbrLYK1b2 — PbrLYK3a2*	Dispersed	\	0.675	2.305	771.03	0.29
	*PbrLYK1b3 — PbrLYK3a2*	Dispersed	\	0.729	1.828	611.36	0.40
LYK2	*PbrLYK2 — PbrLYK4/5a1*	Dispersed	\	0.802	\	\	\
LYK3	*PbrLYK3a1 — PbrLYK3b1*	Dispersed	\	0.642	2.259	755.67	0.28
	*PbrLYK3a2 — PbrLYK3a1*	WGD/segmental	8	0.001	0.021	6.90	0.07
	*PbrLYK3a2 — PbrLYK3b1*	Dispersed	\	0.638	2.073	693.36	0.31
LYK4/5	*PbrLYK4/5b — PbrLYK1a*	Dispersed	\	0.870	\	\	\
	*PbrLYK4/5b — PbrLYK4/5a1*	Tandem	\	0.572	2.616	875.16	0.22
LYM1/3	*PbrLYM1/3–1 — PbrLYM1/3–2*	Dispersed	\	0.605	2.786	931.98	0.22
	*PbrLYM1/3–2 — PbrLYM1/3–3*	Dispersed	\	0.588	2.000	668.94	0.29
LYM2	*PbrLYM2–1 — PbrLYM1/3–2*	Dispersed	\	0.735	1.290	431.63	0.57
	*PbrLYM2–1 — PbrLYM2–2*	WGD/segmental	8	0.009	0.011	3.78	0.77
	*PbrLYM2–2 — PbrLYM1/3–3*	Dispersed	\	0.755	1.529	511.53	0.49
NFP	*PbrNFP1 — PbrNFP2*	Dispersed	\	0.584	\	\	\
	*PbrNFP2 — PbrLYK4/5a1*	Dispersed	\	0.765	\	\	\
	*PbrNFP2 — PbrLYK4/5b*	Tandem	\	0.708	2.581	863.31	0.27

### Conserved motif analysis of the *LYP* gene family in Rosaceae species

The types and composition of inner motifs primarily determine the protein function. To further identify motif construction of the *LYP* gene family in the Rosaceae, the online MEME program was used in this study to detect motif patterns of LYPs. Fifteen conserved motifs with low E values were recognized (Fig. 2b). The number of motifs in LYPs were varied and there were distinctive differences in motif composition between LYM type LYP proteins and other types. The details of each motif and the motif patterns of each subgroup are shown in Supplementary Figs. S2 and S3, respectively. Among Rosaceae LYPs, pattern [#6,14,12,9,6] was shown in almost LYPs as the conserved motifs for the LYP family. However, the pattern [#8,10,7,5,1,3,2,13,4] only could be detected in the KD sequence of LYK type proteins. Without KD, the characteristic motifs were on the N-terminal of LYMs, such as common pattern [#6,14,12,9,6] for LYM1/3 s and partially incomplete pattern [#14,12,9,6] for LYM2s.

Each subfamily had its own relatively certain motif composition with significant differences between LYM and other types of LYPs (Supplementary Fig. S3b), indicating that LYPs are relatively conserved in their evolutionary history and the division among groups may have occurred at an early period. Previous reports have shown that AtLYM2 and AtLYK1/4/5 were all involved in the chitin signal pathway and played a role as core participators or co-receptors to mediate the signaling for their chitin binding ability of the second LysM on the ectodomain [7, 8, 10–13]. In this work, 3 of the 7 conserved residues for chitin binding on that LysM domain were detected on motif #12 (Supplementary Figs. S2 and S3b). This conserved motif was only detected in the extracellular domains of LYK1, LYK4/5, NFP, WAKL, and LYM type groups, which is indicative of inner links between the chitin affinity and the presence of motif #12 and that the evolution between each subgroup may not be completely independent. Interestingly, as the unique motif in LYP family, motif #15 was only detected in LYK3a subfamily (Supplementary Fig. S3b). This indicates that the conserved motif #15 may be related to the opposite function of AtLYK3, as a negative regulator in chitin-induced immunoreactions. Our results suggest that the occurrence of motif #12 and #15 in the ectodomain of Rosaceae LYPs may be related to the chitin affinity and the negative regulation of defensive responses to fungal pathogens, respectively.

### Expression levels of the PbrLYPs

**Fig. 5:**
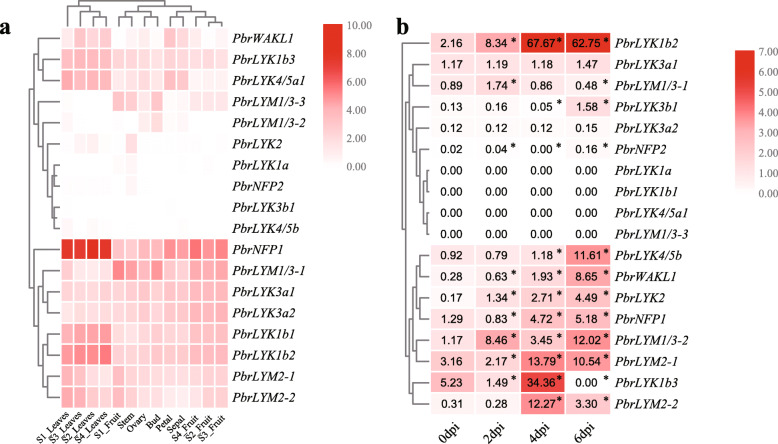
Expression pattern analyses of the *PbrLYP*s in Chinese white pear. **a** Expression analyses of the 18 *PbrLYP*s using previously published transcriptome data in different tissues and stages of Chinese white pear. **b** Expression analyses of the *PbrLYP* genes in leaves by qRT-PCR after *B. dothidea* infection treatments. The pear actin was used as an internal reference for the normalization. Asterisks indicated a significant difference in statistics compared with 0dpi at the indicated time points (* *P* < 0.05)

Previous transcriptome analysis of Chinese white pear revealed tissue-specific expression patterns in petal, sepal, ovary, stem, bud, leaves and fruit [33, 34]. The results indicated that the background expression of most *PbrLYP* genes was rarely detected, however other genes were primarily expressed in fruit and leaves (Fig. 5a). For example, *PbrLYK3a1* and *PbrLYK3a2* were mainly expressed in fruit, petal, sepal and ovary, while *PbrLYK1b3*, *PbrLYK1b1*, *PbrLYK1b2*, *PbrLYK4/5a1* and *PbrNFP1* showed preferential expression levels in leaves. However, *PbrLYM1/3–1* showed highest expression in fruit, stem, and bud, but relatively low expression in leaves.

To verify whether *PbrLYPs* participate in the defense response against *Botryosphaeria dothidea* (*B. dothidea*) pathogen infection, a fungal pathogen that can cause the ring rot disease in apple and pear, we performed an infection treatment experiment with 6-weeks-old pear seedlings. The qRT-PCR (quantitative real-time PCR) results indicated that most of *PbrLYPs* were up-regulated by the infection of *B. dothidea*, with the peak expression occurring at 4 or 6 dpi (Fig. 5b). For example, at 4 dpi, the relative expression of *PbrLYK1b2*, *LYM2–1* and *LYM2–2* was significantly higher than controls at the highest expression. At 6 dpi, the expression levels of *PbrLYK1b2*, *PbrLYK3b1*, *PbrLYK4/5b* and *PbrWAKL1* were still relatively higher than control, as well the peak levels of *PbrNFP2*, *PbrLYK2*, *PbrNFP1* and *PbrLYM1/3–2*. The results indicated that these differentially expressed genes may participate in the defense reactions. However, the expression of *PbrLYK3a1*, *PbrLYK3a2*, *PbrLYK1a*, *PbrLYK1b1*, *PbrLYK4/5a-1* and *PbrLYM1/3–3* showed no significant change following infection in Chinese white pear. Furthermore, the expression of *PbrNFP1*, *PbrLYK1b2*, *PbrWAKL1* and *PbrLYM2–2* was also significantly up-regulated in Qiuzi pear induced by the pathogen infection. On the contrary, the expression levels of *PbrLYM2–1*, *PbrLYK3a2* and *PbrLYM1/3–1* were down-regulated after the infection (Supplementary Fig. S4).

### Subcellular localization of the PbrLYPs

**Fig. 6:**
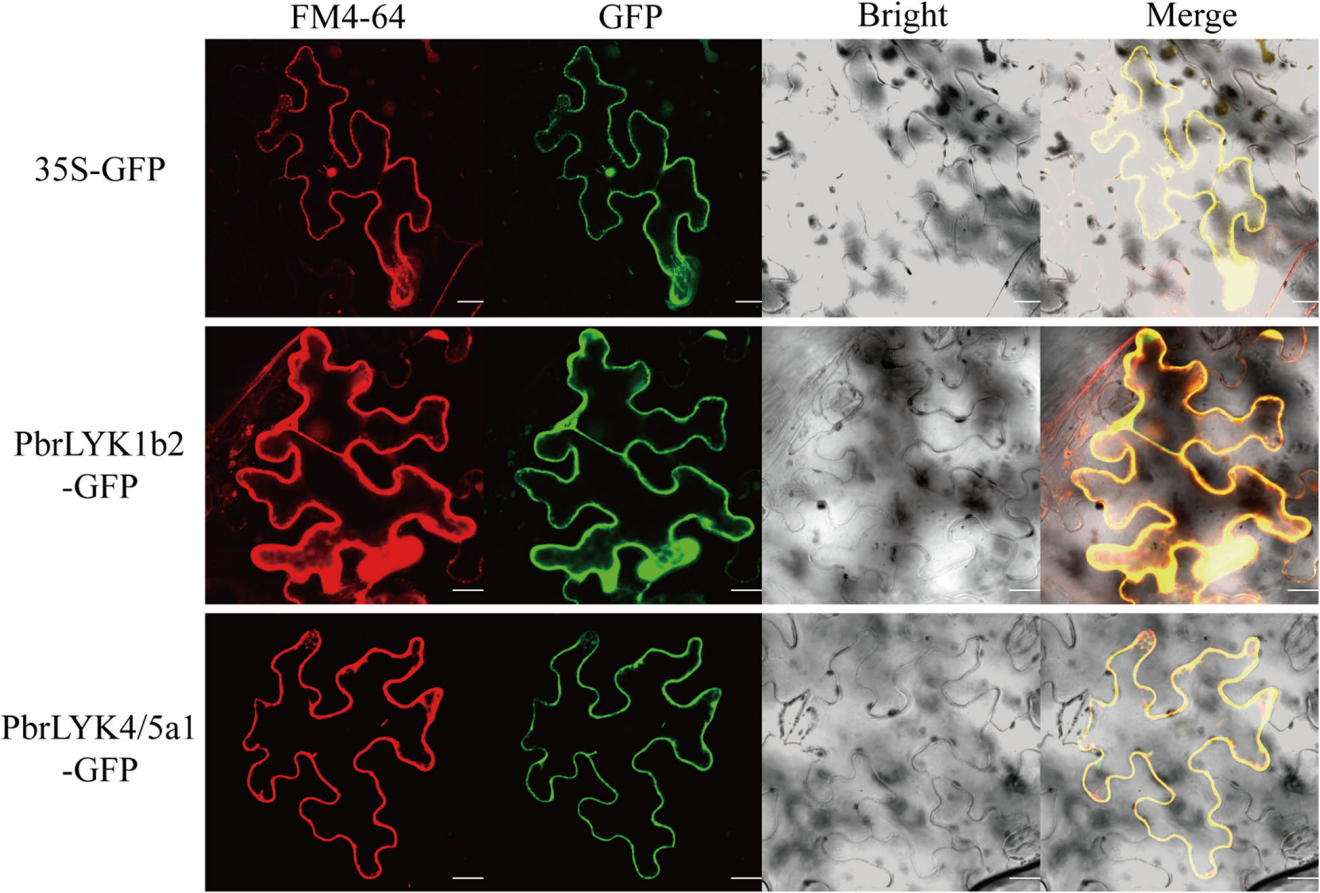
Subcellular localization of PbrLYPs protein. The two fusion proteins and 35S-GFP as the control were expressed transiently in *Nb* leaves in an independent manner and observed via confocal microscope. The merged images include the membrane dye FM4–64 red fluorescence channel (first panels) and green fluorescence channel (second panels). The analogous bright-field images are represented on the right. Bar = 20 μm

PRRs are primarily located in the plasma membrane and are in direct contact with the ligand. To verify whether LYP proteins were also present on the plasma membrane in the Rosaceae and had the potential to act as PRRs, we first performed structural analyses of PbrLYP proteins using the TMHMM online software. The sequence analysis showed that, except for LYM type LYPs, all PbrLYPs contained a transmembrane (TM) region (Supplementary Additional file 3), demonstrating that they can also be localized in the membrane. Considering the effect of signal peptides (SP) on subcellular localization, we selected *PbrLYK1b2* and *PbrLYK4/5a1* to verify the localization of PbrLYPs. The open reading frame of each gene was cloned from pear branches and PbrLYP-35S-GFP fusion proteins or control (35S-GFP alone) were transformed separately into *Nb* leaves. Based on fluorescence microscopy, using the control plasmid, the green fluorescence was found to be scattered in the overall cell. However, *PbrLYK1b2-GFP* and *PbrLYK4/5a1-GFP* containing vectors showed the green fluorescence only in the cell membrane, as depicted in Fig. 6. Therefore, all PbrLYPs with SP and TM seems to have the potential to act as PRRs.

## Discussion

The LysM-containing proteins have been primarily implicated in the PTI immune processes including the early stage of node formation and pathogen perception by direct or indirect PAMP binding. As crucial components of the chitin receptor complex, some members of the *LYP* gene family have been extensively studied in model plants, such as rice and *Arabidopsis*. However, there have been few such efforts to annotate the *LYP* gene family in pear. In this study, we identified 124 *LYP* genes among eight Rosaceae species. In Chinese white pear, 18 *LYP* genes were identified compared to the 21 *LYP* members in apple, while the other Rosaceae species contained 13 to 16 *LYPs*. The number of *LYP* genes in the Rosaceae was much higher than those reported in the model eudicot *Arabidopsis thaliana*. In other words, the expansion of *LYP* family genes in Rosaceae species appeared like a common event. The larger gene numbers in certain LYK1, LYK3 and LYK4/5 groups suggested that these groups may play diverse roles in the adaptive evolution of Rosaceae species to environmental stresses.

The gene duplication analysis showed that the expansion of *LYP* genes in Chinese white pear and apple was primarily due to WGD/segmental events, along with dispersed duplication as the major expansion driving force for *LYPs* in the other six Rosaceae species. According to the widely- spanning Ks values, many large-scale duplication events were detected at the ancient stage (Ks values of 12 of 20 duplicated gene pairs were around 1.290 ~ 2.786) in Chinese white pear (Table 4). The results suggested that the selection of the function of perception and defense response to chitin was beginning at a very early stage and continuing up to now. These functions are fundamental and vital for plant survival. The *LYPs* in LYK1/3/4/5 and LYM2 groups were reported to be closely related to chitin signaling [7, 8, 10–13]. In this study, six out of seven WGD/segmental-type *PbrLYPs* were detected in LYK1, LYK3 and LYM2 groups, suggesting that the evolution of chitin response was mainly derived from the WGD/segmental events and remained in Chinese white pear. The Ka/Ks ratios of all duplicated *PbrLYP* pairs were less than one, which implied that the *PbrLYPs* are undergoing purifying selection and they seem to be necessary for adaptation to the current environment in their evolutionary history.

Phylogenetic analysis classified the Rosaceae *LYPs* into eight subgroups, which suggested that the evolution of different subfamilies was relatively independent. Analysis of the gene structure and protein motif showed the high similarity of the motif composition and exon-intron architecture within each subgroup also confirming independent evolution (Fig. 2b, c). The above results suggested that the genes in the same clade may have similar evolutionary histories and may perform a similar function. As shown in the gene structure analyses, subfamilies LYK1 and LYK3 contained the highest number of exons in LYKs, indicating that intronization in the exons of the genes (in these groups) might have happened. The number of exons also has a key contribution to their divergent functionality in various tissues, organs, or growth periods. A similar case was also found in the LYM2 group in the LYMs in European pear, strawberry, and black raspberry.

According to the previous works about the evolution of the plant *LYP* gene family, the *LYPs* have evolved through local and segmental duplications and can be grouped into three main types: LYP-I (about 10 exons per gene and containing conserved KD), LYP-II (one to five exons per gene, lacking the KD), and LYP-III (one or two exons per gene, with a KD unlike that of LYP-I), likely arising from the fusion of other type *LYP* genes [16, 35–37]. The LYP-I type gene products are the main PRRs in each signaling pathway. LYP-II types are likely to not function as core receptor kinases, but form complexes with other LYPs, such as that AtLYK1 that could interact with AtLYM1/3 and AtLYK4/5 to mediate bacterial and fungal pathogen perception in *Arabidopsis*, respectively [7, 11, 12, 38]. The Rosaceae *LYPs* were well-matched to the characteristics in protein and gene structure of *AtLYPs*, and therefore may potentially have similar roles in signaling. With the higher number of genes and exons, the genes in the LYM2 group of sweet cherry and black raspberry and genes in LYK1 and LYK3 groups of other species seemed to have undergone stronger evolutionary selection and may be more diverse in function. In addition, we also investigated the conserved motifs of LYPs and determined the putative protein localization as well as their collinearity relationships. In total, 15 distinct conserved motifs among various LYP proteins were predicted by the MEME analysis. As shown above in Fig. 2b and Fig. S3b, motif patterns [#6,14,12,9,6] and [#8,10,7,5,1,3,2,13,4] might represent the functional motifs of ectodomain and intracellular kinase domain of LYPs, respectively. Meanwhile, a LYK3a-unique motif #15 was detected in the region of the juxtamembrane domain in the LYK3a protein group, where it could be regulated by phosphorylation to affect the activity of the kinase domain [39]. The AtLYK3 was also placed into LYK3a group with the motif #15. Therefore, it is reasonable to consider that the motif #15 was related to the negative regulatory functions of the genes in LYK3a. However, this question requires further research. The conservative residues for chitin binding were detected in the motif #12, and that motif was only found in LYK1/4/5, NFP, WAKL and LYM type groups (Fig. S3b), suggesting that the genes in those groups likely shared a common ancestor and had the similar functions at the ancient period in response to chitin. After a long period of evolution and selection, the duplicated Rosaceae *LYPs* remained in relatively large numbers, suggesting that the *LYP* genes were important for Rosaceae species in adaptation to the complex and changing environments.

The *LYP* gene family plays various important roles in growth and response to biotic stresses. For example, *AtLYK1* encodes a plasma membrane-localized receptor kinase protein. AtCERK1 works as a receptor homodimer or the core element of the hetero-tetramer with AtLYK4/5 or AtLYM1/3. These complexes are involved in initiating PTI responses against the fungal or bacterial pathogen infection in *Arabidopsis* [7, 8, 10–12, 38]. Transcriptome data showed that in the common target tissues for pathogen infection, such as leaves and fruit, some *PbrLYPs* had relatively higher expression than in other tissues for host protection. Based on the expression patterns, these *PbrLYPs* may be regarded as putative defense-related genes at the background level. In China, fruit ring rot and stem wart diseases caused by pathogen *B. dothidea* occur in almost all pear-growing areas, and the target organs including pear fruit, stem, shoots and leaf [40]. Although the pathogenesis of *B. dothidea* was poorly understood, the previous work in apple had reported that a LysM-containing protein gene, *MdCERK1–2*, was involved in the anti-fungal defense responses as a PRR and significantly upregulated after *B. dothidea* infection [23]. In other word, the chitin signaling pathway was likely recruited during the infection of *B. dothidea*. To verify whether *PbrLYPs* were involved in the defense reaction, we performed an infection treatment and qRT-PCR analysis. Our qRT-PCR results indicated that some of *PbrLYPs* participated in the immune response to *B. dothidea* infection (Fig. 5 and Fig. S4). In addition, after the infection with *B. dothidea*, significantly increased relative expression of several putative defense-related genes was detected by qRT-PCR, including *PbrLYK1b2*, *PbrNFP1*, *PbrWAKL1* and *PbrLYM2–2*, which is consistent with the case of *MdCERK1–2* in apple [23]. It is interesting to note that, although the expression level of *PbrLYK4/5b* in Chinese white pear was strongly up-regulated by the fungal pathogen infection, it could not be detected in Qiuzi pear before or after infection. This may have been due to the relatively high expression levels of *PbrLYK1b1*, *PbrLYK1b2* and *PbrWAKL1* in Qiuzi pear compared to Chinese white pear. Therefore, if some of the LYPs were the core PRR of chitin perception complex and able to form a homodimer, like in *Arabidopsis*, then these extensively expressed proteins may perform full functions independently activate the chitin signaling pathway. Hence, there may be no need to recruit co-receptors like PbrLYK4/5b to form a recognition complex, possibly accounting for the high pathogen resistance of Qiuzi pear. This question requires further investigation to reveal the infection strategy of *B. dothidea* and the resistant mechanism of host pear plant. Furthermore, the subcellular localization analysis demonstrated that pathogen-inducible genes (PbrLYK1b2 and PbrLYK4/5b) were also located at the plasmalemma, suggesting a potential capacity for PbrLYKs to act as PRRs at the subcellular level. In addition to the expression analysis, these results were consistent with previous studies that have implicated *LYP* genes in biotic stress tolerance via chitin-binding chitin and activation of the downstream immune response as plasmalemma-located PRRs [7, 11, 12, 38].

However, further investigation will be required to determine whether the expansion of LYPs could provide more advanced pathogen detection model to increase the chances of surviving under the complex environmental changes and the receptor complex in Chinese white pear or other Rosaceae species similar to that in *Arabidopsis* or rice. The characterization of key elements and the composite pattern of these complexes was also crucial to the understanding of the functional mechanisms of LYPs in the Rosaceae.

## Conclusions

One hundred twenty-four full-length *LYP* genes were determined in the eight genomes of Rosaceae, along with the 18 *LYP* genes of the Chinese white pear genome. Based on the protein sequences and CDS structural characteristics, comparison with *Arabidopsis* homologs, and phylogenetic analysis, the *LYP* genes were classified into eight groups i.e.*,* LYK1, LYK2, LYK3, LYK4/5, LYM1/3, LYM2, NFP, and WAKL, with groups LYK1 and LYK3 possibly having higher functional diversity. According to the analysis of collinearity, the ancient and recent WGD and dispersed duplication might have a role in the evolution of the *LYP* gene family, associated with apple and Chinese white pear. The LYP family genes were found to be greatly influenced via evolutionary negative selection. qRT-PCR revealed that *LYP* genes might have a vital role against the fungal pathogenesis. The underlined collected data establish a foundation for advanced studies to evaluate the complexity of *LYP* gene family in the Rosaceae.

## Methods

### Determination of *LYP* genes in Chinese white pear and other species of Rosaceae

For the determination of the *LYP* genes in pear and other species of Rosaceae, several databases were employed. To acquire LYP family genes, we used the following strategy: The genome sequences of eight species belong to Rosaceae were downloaded from each genome project (Supplementary Table S3). Subsequently, we built a Hidden Markov Model (HMM) with the extracellular domain sequences of 12 well-studied LYP proteins (AtLYK1–5, AtLYM1–3, OsCERK1, OsCEBiP, OsLYP4 and OsLYP6, the accession numbers and extracellular domain sequences were shown in Table S5) [41], using the HMMER3 software package [42, 43], and downloaded the seed file of Lysin Motif domain (PF01476) from the Pfam database (http://pfam.xfam.org/). The sequences of eight *Arabidopsis* proteins and four rice proteins were acquired from TAIR (https://www.arabidopsis.org/) and NCBI (https://www.ncbi.nlm.nih.gov/), respectively. Then HMM searches with PF01476 and self-build model were independently conducted for the local protein databases of eight species of Rosaceae via HMMER3 with E-values <1e^− 10^. Furthermore, two resulting gene lists were intersected and the protein sequences were detected by the NCBI Batch CD-Search tools (Batch CD-Search: https://www.ncbi.nlm.nih.gov/Structure/bwrpsb/bwrpsb.cgi) based on CDD v3.18 and SMART v6.0 databases for the validation of the existence of the LysM domain. The sequences of proteins with E-values greater than 1e^− 6^ or without a LysM domain were deleted. The relevant accession numbers of *LYP* genes were shown in Table 1.

### Structure and conserved motif analysis of the *LYP* genes

The Gene Structure Display Server (GSDS 2.0) (http://gsds.cbi.pku.edu.cn/) was used to analyze the structures of the *LYP* genes by aligning the cDNA sequences with their corresponding genomic DNA sequences. Conserved motif analysis of LYP proteins was performed by online Multiple Expectation Maximization for Motif Elicitation (MEME) (http://meme.nbcr.net/meme/cgibin/meme.cgi) [44]. Maximum number parameter of motifs was seted as 15.

### Phylogenetic analysis

The construction of phylogenetic trees was carried out with Neighbor-Joining (NJ) and a bootstrap of 1000 in MEGA7.0 (http:// www.megasoftware.net/) [45]. The p-distance was used and the optional parameters for pairwise deletion were considered.

### Chromosomal localization and synteny analysis

Genome annotation files of *Arabidopsis* and eight Rosaceae species were downloaded from TAIR and each genome project (Supplementary Table S3). The same procedure used in the PGDD (http://chibba.agtec.uga.edu/duplication/) [46] was performed to analyze the synteny among the LYPs. Primarily, for the investigation of considerable pairs of the homologous gene, the local all-vs-all BLASTP searches among *Arabidopsis* and eight species belong to Rosaceae genomes were conducted (E < 1e^− 10^). Afterward, MCScanX was employed for the determination of syntenic gene pairs with the BLASTP result and gene location information used as input files [47]. The downstream analysis tool (duplicate_gene_classifier) in the MCScanX package was employed for the identification of tandem, proximal dispersed, and segmental/whole-genome duplications (WGD) of LYP family genes. The results were visualized using circos-0.69 software [48]. The Ka and Ks values were analyzed via KaKs_Calculator 2.0 [49]. For the estimation of the date of segmental duplication events, the succeeding pairs of homologous genes within 100 Kb on all sides of the *LYP* genes, considered for the mean Ks calculation.

### Subcellular localization of the PbrLYPs

The amplification of total-length CDS of the *PbrLYK1b2* and *PbrLYK4/5a1* was carried out via PCR, respectively. Purified products were subcloned directionally into the modified *pCAMBIA1300-GFP* vector (Clontech, Beijing, China), and resulted in *PbrLYK1b2-GFP* and *PbrLYK4/5a1-GFP*. Primers assisting gene cloning and vectors construction, depicted in added Table S4. The agrobacterium carrying above products were transformed into 4-week-old *Nb* leaves, respectively, as the method reported previously with slight modification [50]. Images were obtained via the Zeiss LSM Image Browser (Zeiss LSM 780, Germany). The independent assays were conducted at a minimum of thrice for each gene. The empty vector *pCAMBIA1300-GFP* was used as control.

### Infection treatment and quantitative real-time PCR

Chinese white pear (Dangshansuli, *Pyrus bretschneideri* Rehd.) and “Qiuzi” pear (*Pyrus ussuriensis* Maxim) seeds were obtained from the pear germplasm orchard of the Center of Pear Engineering Technology Research situated at Hushu in Nanjing and were allowed to grow in soil pots in a maintained environment (2:1 light/ dark period, 25 °C) in the phytotron. Sixty leaves of each kind of pears were harvested from 15 six-week-old seedlings and placed on the sterile water wetted filter paper in a petri dish overnight. Then the 5-day-old fresh *B. dothidea* mycelia, which grown in the PDA plat, were stuck to the paraxial surface of leaves to perform infection. The infected leaves were cryopreserved with liquid nitrogen at 0 dpi (day post infection), 2dpi, 4dpi, and 6dpi. Total RNA extraction and the synthesis of cDNA were according to the instructions of RNA kit (Tiangen, Beijing, China) and PrimeScript RT reagent Kit (Trans Gen). Specialized primers of the constitutive *TUB* and *PbrLYP* genes were designed via NCBI online tool Primer-BLAST (https://www.ncbi.nlm.nih.gov/tools/primer-blast/index.cgi? LINK_LOC=BlastHome) with the Specificity Parameters Organism option set as *Pyrus bretschneideri* (taxid:225117) (Supplementary Table S4). The specificity of each primer pairs was verified by the online program Primer search-Paired against the pear genome. The qRT-PCR assays were conducted with three technical copies. QRT-PCR reactions (20 μl per hole) were performed as previously reported [51]. The expression was evaluated for each sample via the 2^−ΔΔCt^ method, and Duncan’s multiple range test was conducted. A *P-*value of less than 0.05 was the considerable variation and indicated with asterisks. The reported RNA-seq data was processed for the evaluation of the expression patterns of *PbrLYPs* (obtained from the NCBI bioproject PRJNA563942 and PRJNA498777) [33, 34], the differentially expressed genes were identified with |log2FC| > 1. The heatmaps were drawn in TBtools v0.666 [52].

## Supplementary information

**Additional file 1: Table S1.** Exon number statistical analysis of *LYP* genes. **Table S2.** Duplication type of *LYP* genes in *Arabidopsis* and eight Rosaceae species. **Table S3.** Genome information of eight Rosaceae species. **Table S4.** Primers of *PbrLYPs* for qRT-PCR and vector construction. **Table S5.** Extracellular domain sequences of 12 LYP proteins from *Arabidopsis* and rice.

**Additional file 2: Fig. S1.** Gene position of *PbrLYPs*. The characters in blue indicate the chromosome or scaffold number. The height of columns in red indicate the length of chromosome. The number on the right side of the chromosome indicate the start position of each gene. **Fig. S2** 15 MEME motifs of LYPs. Over-represented motifs in *Arabidopsis* and the eight Rosaceae species were identified using the MEME tool. The stack’s height indicates the level of sequence conservation. The heights of the residues within the stack indicate the relative frequencies of each residue at that position. The star symbols under Motif 12 indicate the conservative positions for chitin binding. **Fig. S3.** Schematic diagram and distribution of conserved motifs among the LYP proteins of *Arabidopsis* and eight Rosaceae species. a: The schematic diagram of conserved motifs among the LYP proteins detected by NCBI Batch CD-Search. 4EBZ: the ectodomain of AtCERK1 in PDB databases (https://www.rcsb.org/). b: The distribution of conserved motifs among the LYP proteins detected by MEME. The red and black squares represent non- and conservative MEME domains in the subfamily. The blue square represents the unique motif in LYK3a subgroup. **Fig. S4** qRT-PCR analyses of the *PbrLYP* genes in Qiuzi pear leaves after *B. dothidea* infection. The pear actin was used as internal reference for the normalization. Asterisks indicated significant difference in statistics compared with 0dpi at the indicated time points (* *P* < 0.05).

**Additional file 3.** Transmembrane regions prediction of AtLYPs and PbrLYPs.

## Data Availability

The relevant accession numbers of all *LYP* genes from eight used Rosaceae species were obtained from each genome annotation files and shown in Table 1: All needed genome sequences and genome annotation files of Chinese white pear, *Arabidopsis* and Mei were obtained from the Nanjing Agricultural University pear genome project website (http://peargenome.njau.edu.cn), The Arabidopsis Information Resource (TAIR, https://www.arabidopsis.org/) and a clone of the genome data of the *Prunus mume* genome project website from GitHub (https://github.com/lileiting/prunusmumegenome), respectively; all needed genome sequences and genome annotation files of other six used Rosaceae species were downloaded from the Genome Database for Rosaceae (European pear: ftp://ftp.bioinfo.wsu.edu/species/Pyrus_communis/Pcommunis-draft_genome.v1.0/; Apple: ftp://ftp.bioinfo.wsu.edu/species/Malus_x_domestica/Malus_x_domestica-genome_GDDH13_v1.1; Strawberry: ftp://ftp.bioinfo.wsu.edu/species/Fragaria_vesca/Fvesca-genome.v4.0.a2/; Peach: ftp://ftp.bioinfo.wsu.edu/species/Prunus_persica/Prunus_persica-genome.v2.0.a1/; Sweet cherry: ftp://ftp.bioinfo.wsu.edu/species/Prunus_avium/Prunus_avium-genome.v1.0.a1; Black rasberry: ftp://ftp.bioinfo.wsu.edu/species/Rubus_occidentalis/Rubus_occidentalis-genome.v3.0/). The sequences of the 12 well-studied LYP proteins (AtLYK1–5, AtLYM1–3, OsCERK1, OsCEBiP, OsLYP4 and OsLYP6) were acquired from TAIR and NCBI (National Center for Biotechnology Information, https://www.ncbi.nlm.nih.gov/), respectively, and the accession numbers of them were listed in Table S5. The seed file of Lysin Motif domain (PF01476) were downloaded from the Pfam database (http://pfam.xfam.org/). The transcriptome raw reads from different pear tissue and stage have been deposited at NCBI (https://www.ncbi.nlm.nih.gov/bioproject/) under accession numbers PRJNA563942 and PRJNA498777.

## Abbreviations

LYP: Lysin motif containing protein
LYK: LysM receptor kinase
LYM: LysM-containing glycosylphosphatidylinositol-anchored protein
LysM: Lysin motif
RLK: Receptor-like kinase
NFP: Nod factor perception protein
WAKL: Wall associated kinase-like
WGD: Whole-genome duplication
MYA: Million years ago
PAMP: Pathogen-associated molecular pattern
PRR: Pattern-recognition receptor
PTI: PAMP-triggered immunity
EF-Tu: Elongation factor Tu
flg22: Bacterial flagellin epitope
FLS2: Flagellin sensing 2
KD: Kinase domain
kd: Dissociation constant
ROS: Reactive oxygen species
MAPK: Mitogen-activated protein kinase
PR: Pathogenesis-related
CEBiP: Chitin elicitor binding protein
CERK: Chitin elicitor receptor kinase
PI: Protein isoelectric point
GRAVY: Grand average of hydropathy
Ka: Nonsynonymous substitutions per site
Ks: Synonymous substitutions per site
*B. dothidea*: *Botryosphaeria dothidea*
qRT-PCR: Quantitative real-time Polymerase Chain Reaction
CDS: Coding sequence
*Nb*: *Nicotiana benthamiana*
dpi: Day post infection
