# Supplementary material for: Genome-wide identification of lysin motif containing protein family genes in eight rosaceae species, and expression analysis in response to pathogenic fungus Botryosphaeria dothidea in Chinese white pear
Source: BMC Genomics. 2020 Sep 7;21:612. doi: 10.1186/s12864-020-07032-9 (PMC7487666; doi:10.1186/s12864-020-07032-9)
Supplement: Supplementary file 2 — Additional file 2: Fig. S1. Gene position of PbrLYPs. The characters in blue indicate the chromosome or scaffold number. The height of columns in red indicate the length of chromosome. The number on the right side of the chromosome indicate the start position of each gene. Fig. S2 15 MEME motifs of LYPs. Over-represented motifs in Arabidopsis and the eight Rosaceae species were identified using the MEME tool. The stack’s height indicates the level of sequence conservation. The heights of the residues within the stack indicate the relative frequencies of each residue at that position. The star symbols under Motif 12 indicate the conservative positions for chitin binding. Fig. S3. Schematic diagram and distribution of conserved motifs among the LYP proteins of Arabidopsis and eight Rosaceae species. a: The schematic diagram of conserved motifs among the LYP proteins detected by NCBI Batch CD-Search. 4EBZ: the ectodomain of AtCERK1 in PDB databases (https://www.rcsb.org/). b: The distribution of conserved motifs among the LYP proteins detected by MEME. The red and black squares represent non- and conservative MEME domains in the subfamily. The blue square represents the unique motif in LYK3a subgroup. Fig. S4 qRT-PCR analyses of the PbrLYP genes in Qiuzi pear leaves after B. dothidea infection. The pear actin was used as internal reference for the normalization. Asterisks indicated significant difference in statistics compared with 0dpi at the indicated time points (* P < 0.05). [file 12864_2020_7032_MOESM2_ESM.pdf]

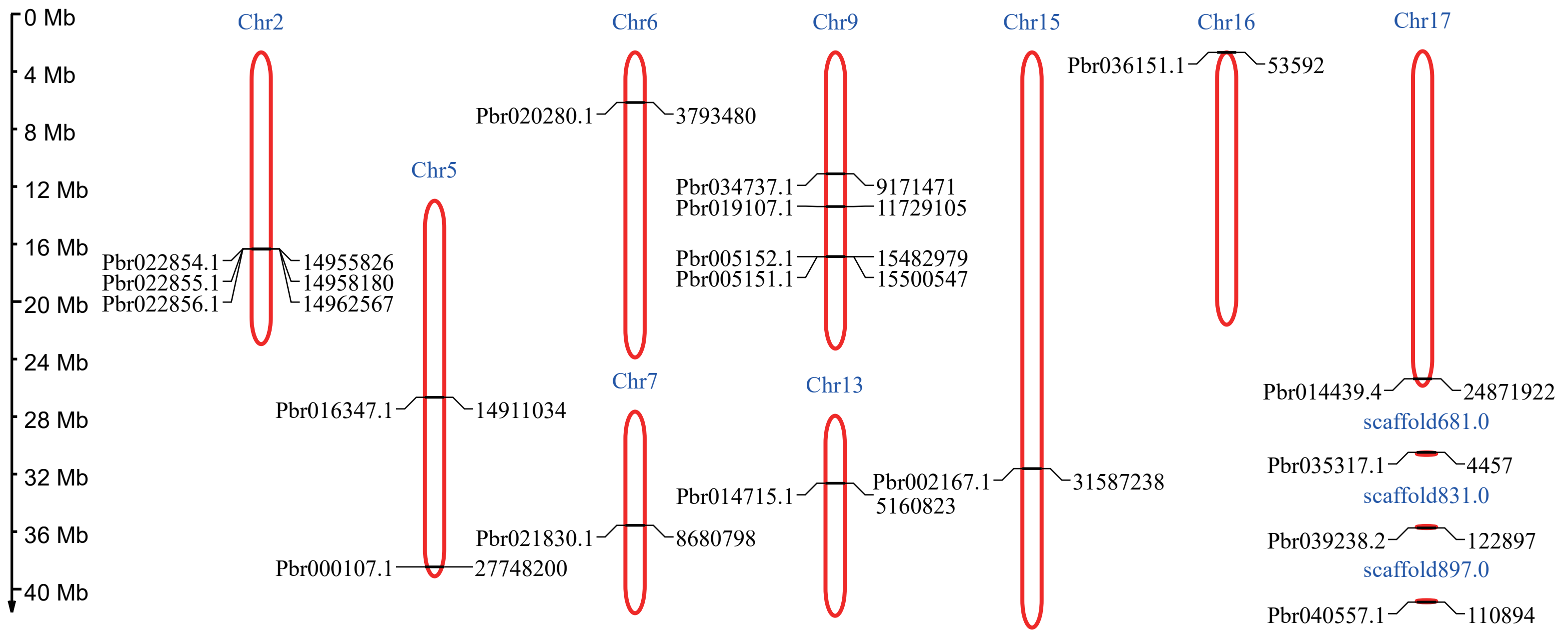


Fig S1 Gene position of *PbrLYPs*.

The characters in blue indicate the chromosome or scaffold number. The height of columns in red indicate the length of chromosome. The number on the right side of the chromosome indicate the start position of each gene.

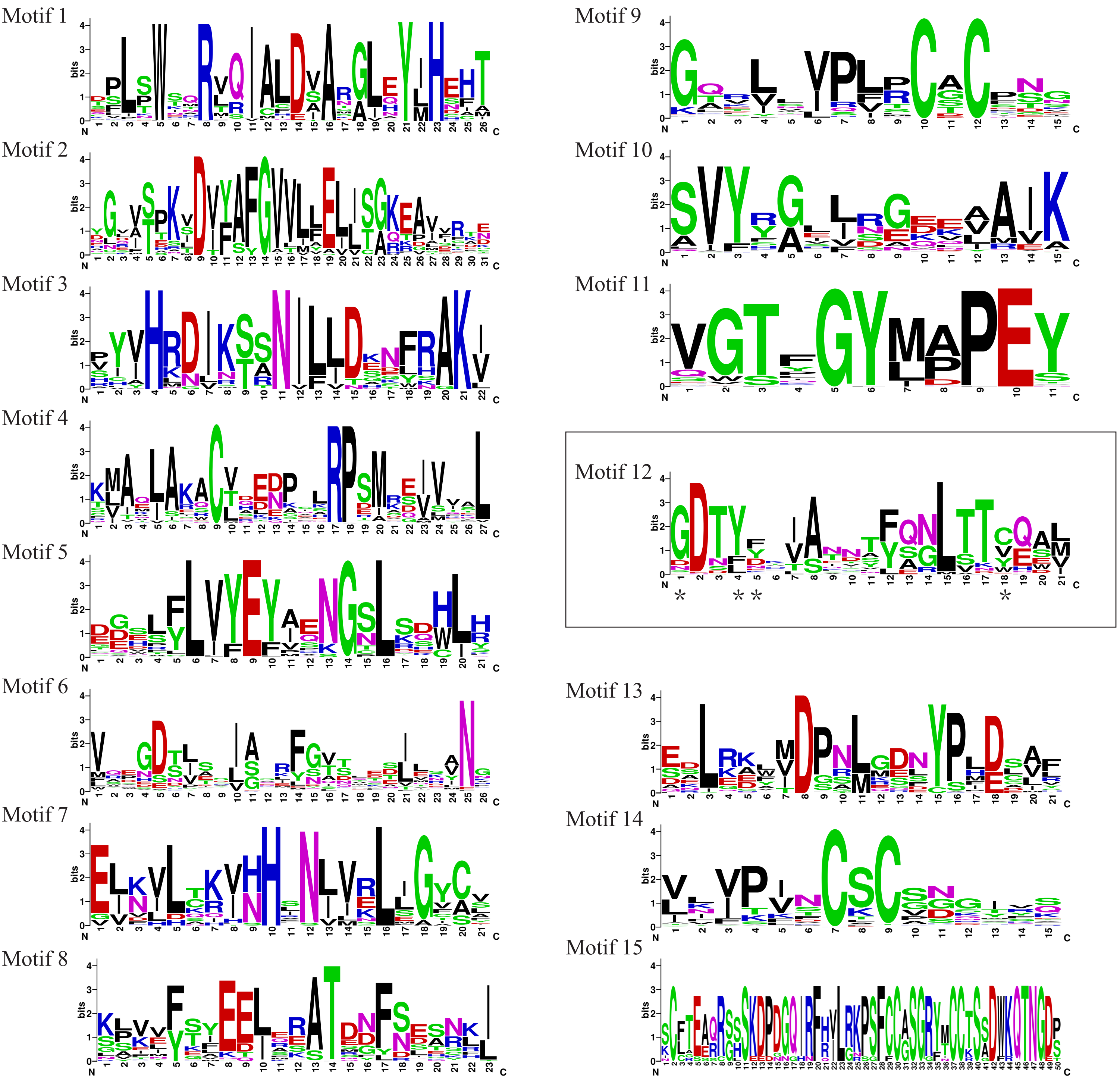


Fig S2 15 MEME motifs of LYPs.

Over-represented motifs in Arabidopsis and the 8 Rosaceae species were identified using the MEME tool. The stack's height indicates the level of sequence conservation. The heights of the residues within the stack indicate the relative frequencies of each residue at that position. The star symbols under Motif 12 indicate the conservative positions for chitin binding.

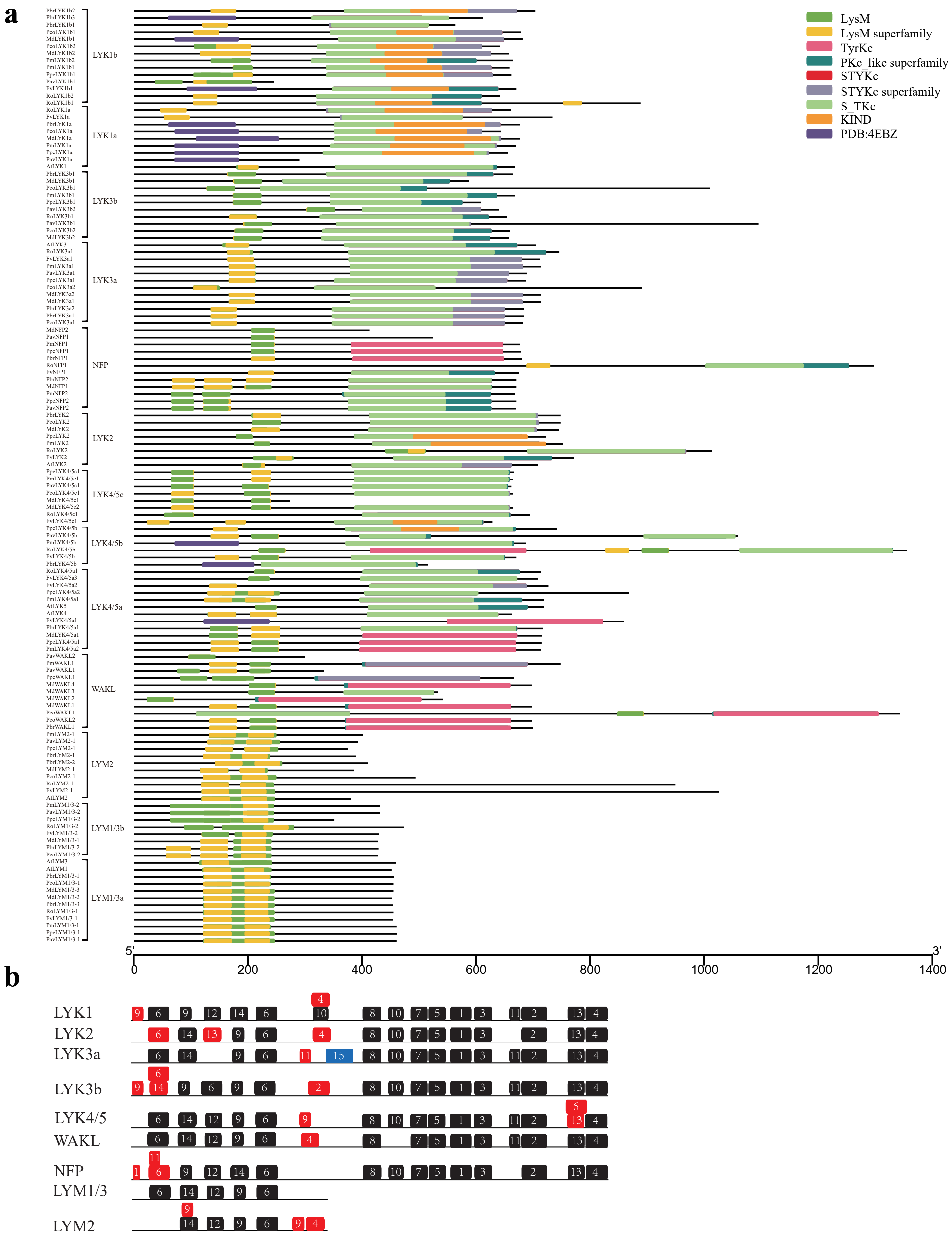


Fig S3 Schematic diagram and distribution of conserved motifs among the LYP proteins of *Arabidopsis* and 8 Rosaceae species.

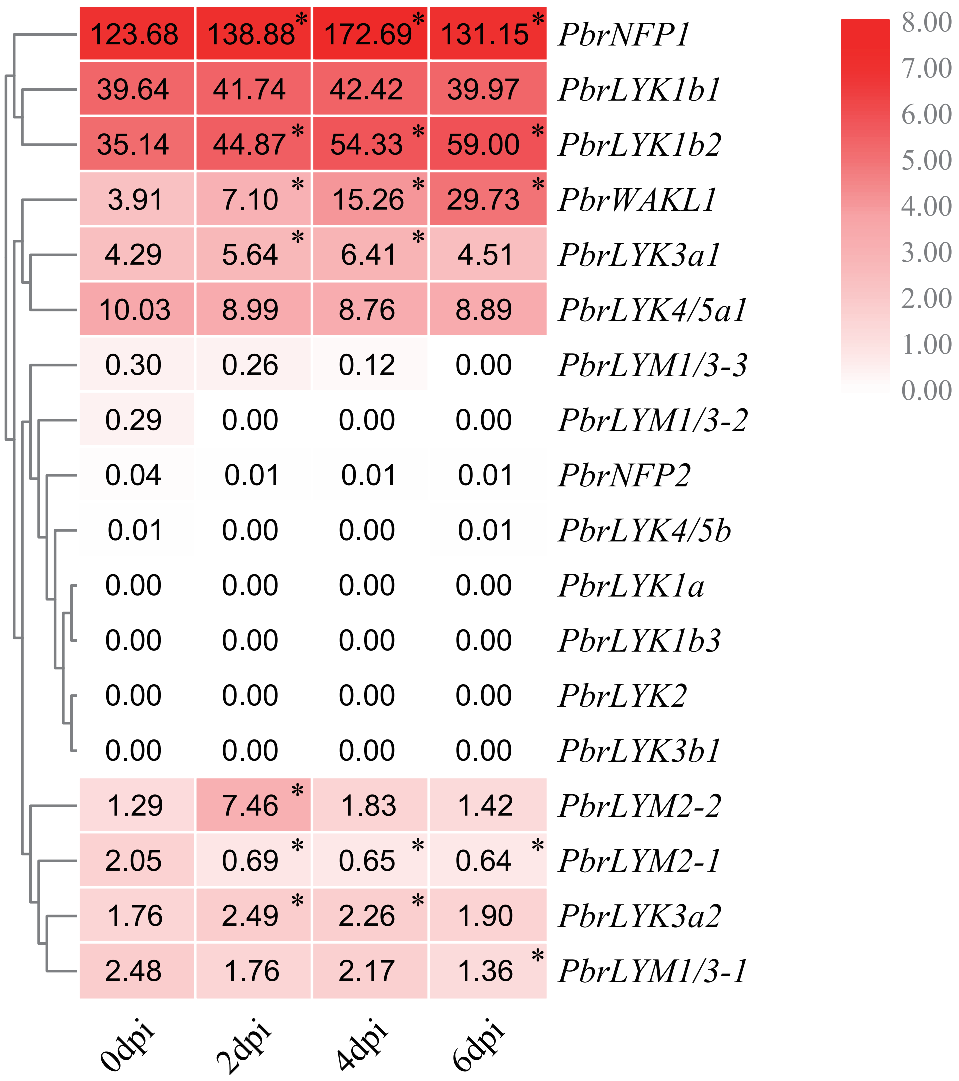


Fig S4 qRT-PCR analyses of the PbrLYP genes in Qiuzi pear leaves after *B. dothidea* infection. The pear actin was used as internal reference for the normalization. Asterisks indicated significant difference in statistics compared with 0dpi at the indicated time points (* $P < 0.05$).
