# Supplementary material for: Genome-wide identification of lysin motif containing protein family genes in eight rosaceae species, and expression analysis in response to pathogenic fungus Botryosphaeria dothidea in Chinese white pear
Source: BMC Genomics. 2020 Sep 7;21:612. doi: 10.1186/s12864-020-07032-9 (PMC7487666; doi:10.1186/s12864-020-07032-9)
Supplement: Supplementary file 3 — Additional file 3. Transmembrane regions prediction of AtLYPs and PbrLYPs. [file 12864_2020_7032_MOESM3_ESM.pptx]

## Slide 1
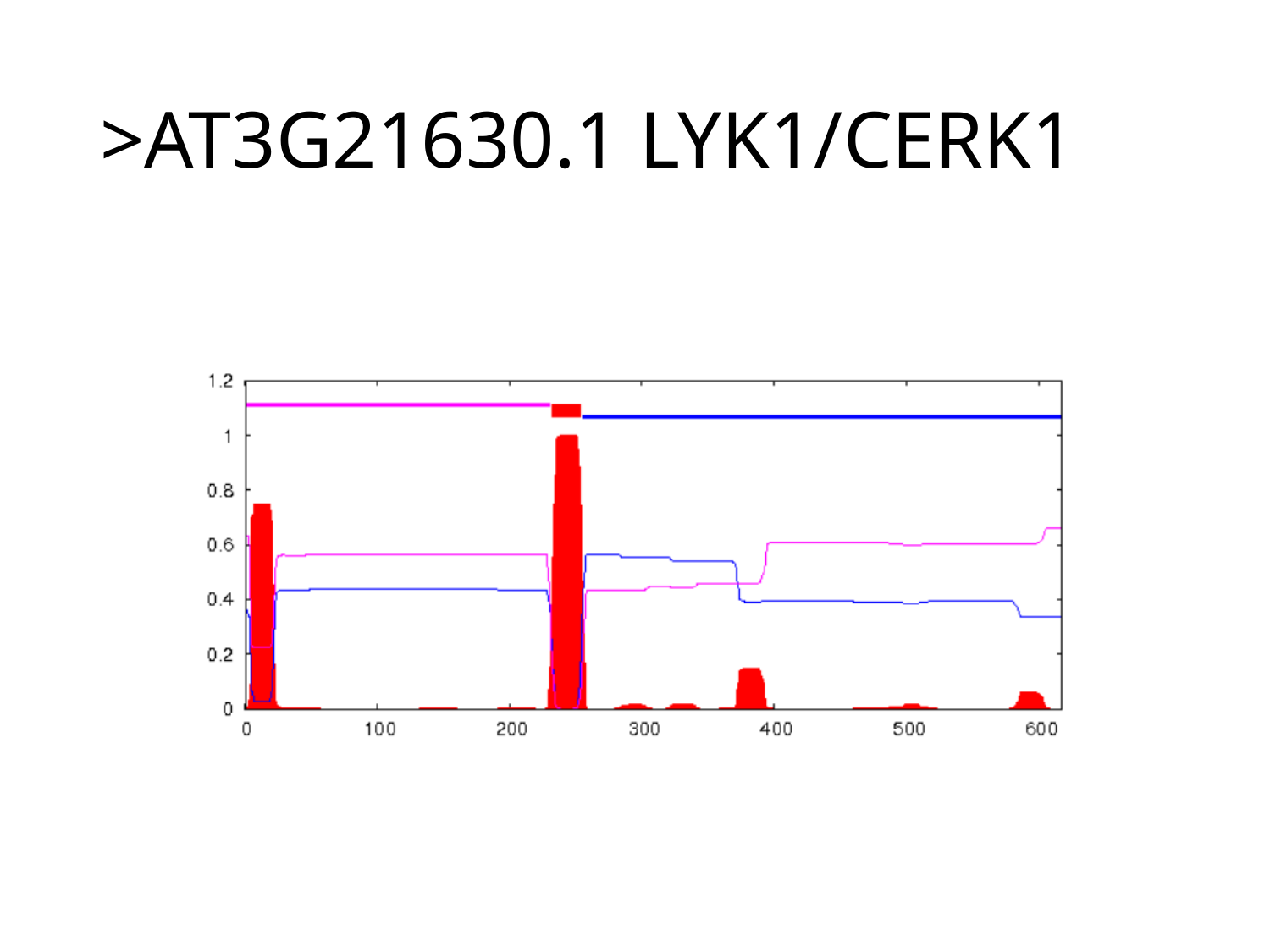

# >AT3G21630.1 LYK1/CERK1

## Slide 2
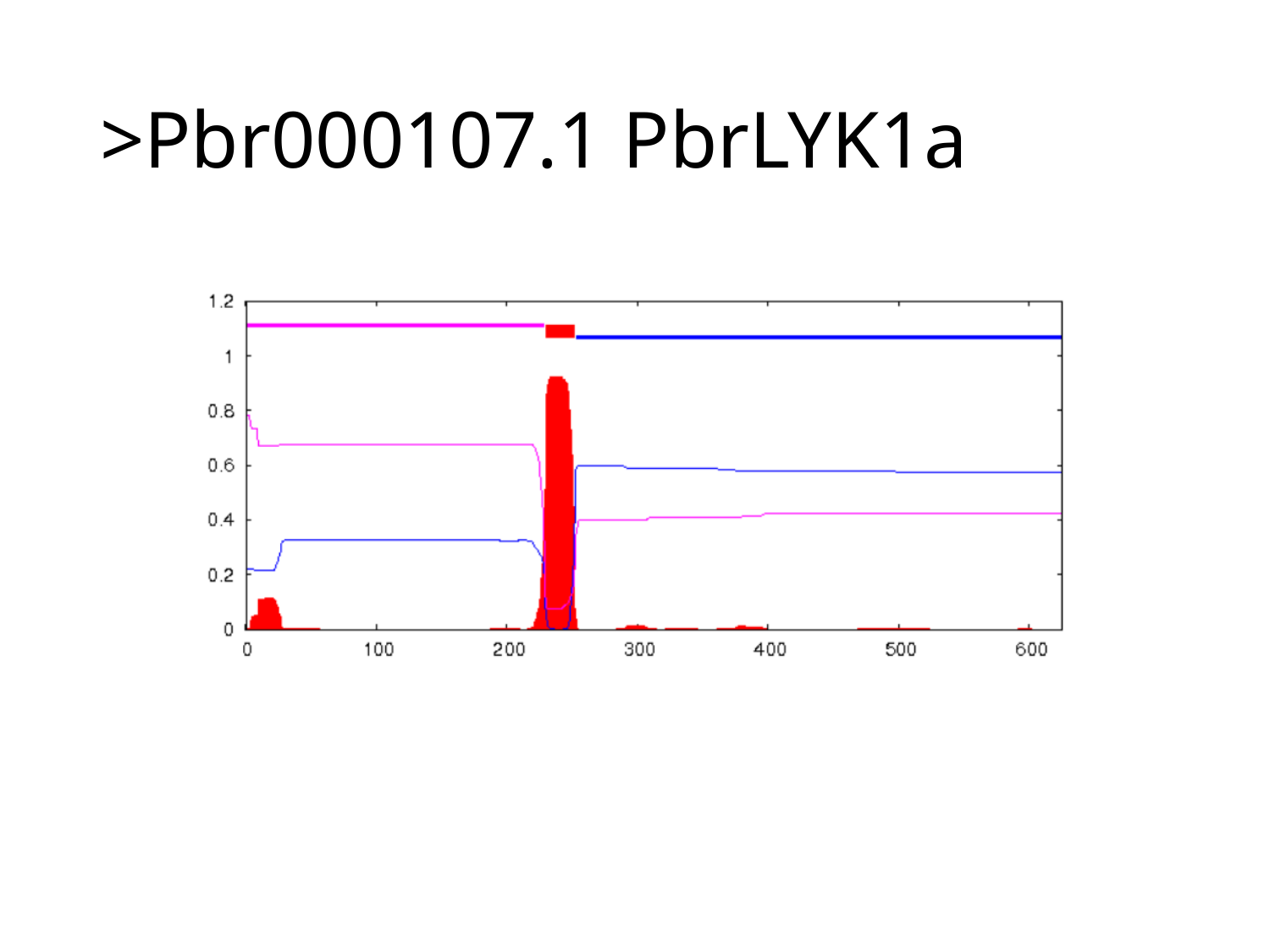

# >Pbr000107.1 PbrLYK1a

## Slide 3
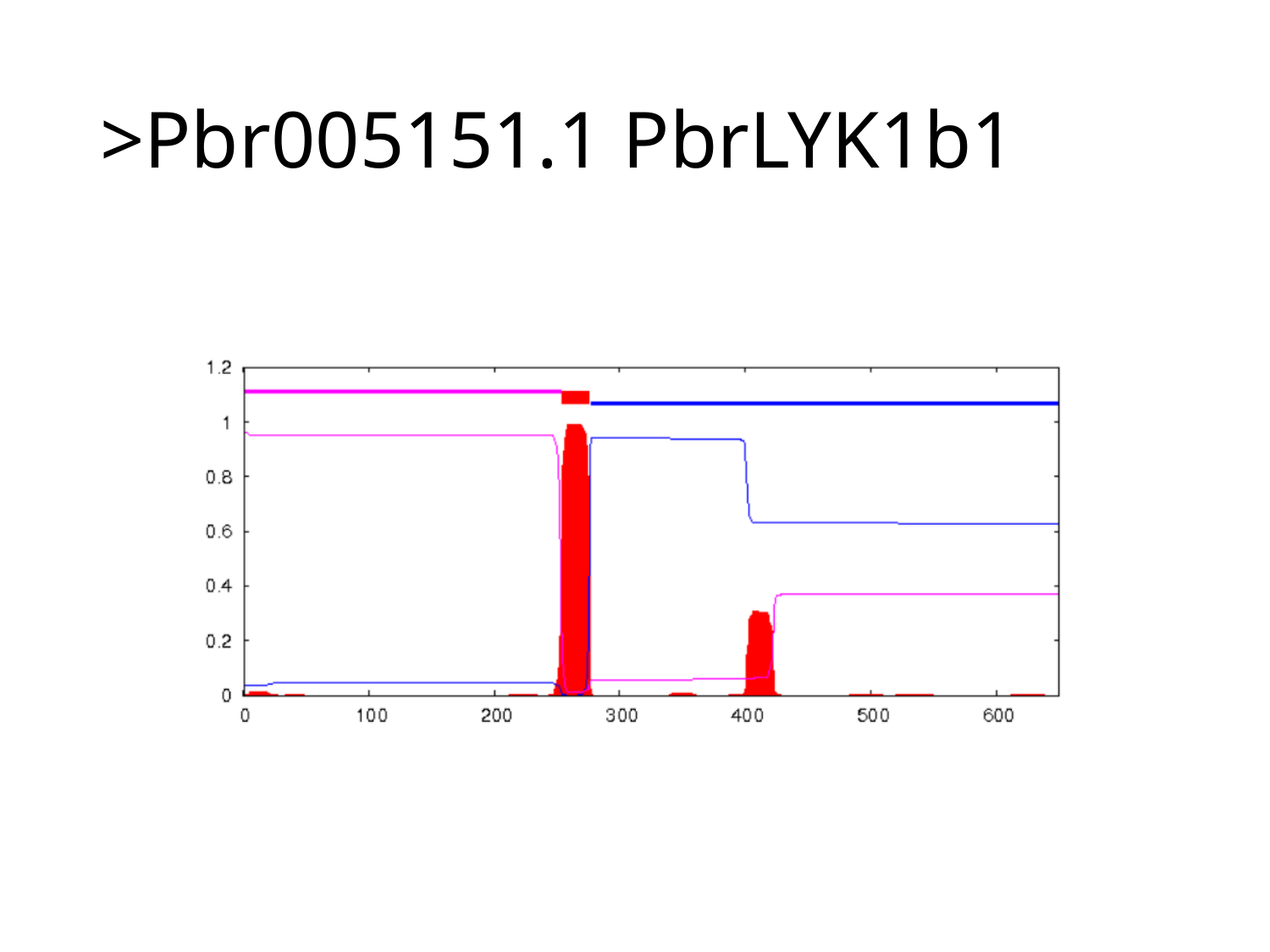

# >Pbr005151.1 PbrLYK1b1

## Slide 4
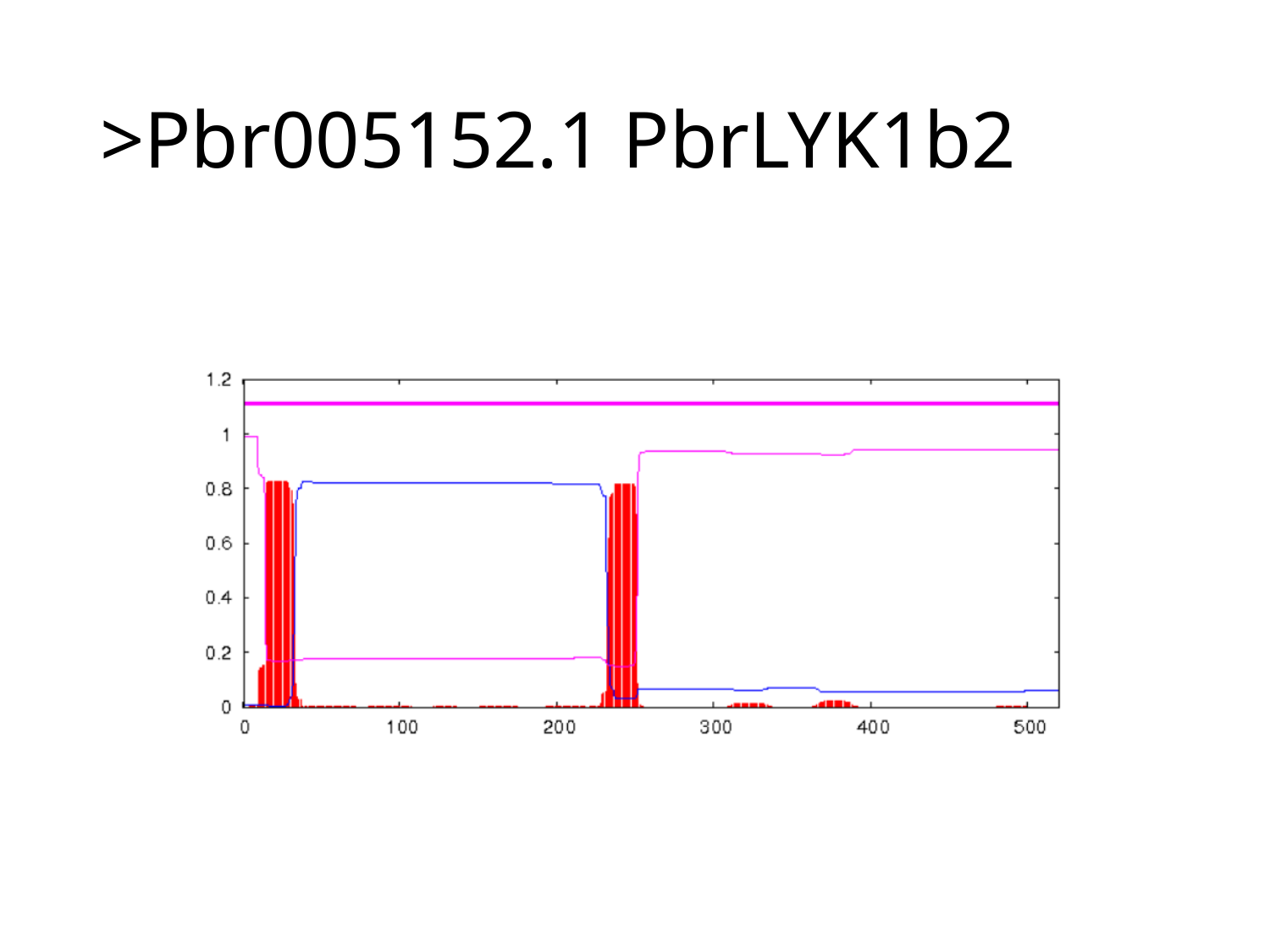

# >Pbr005152.1 PbrLYK1b2

## Slide 5
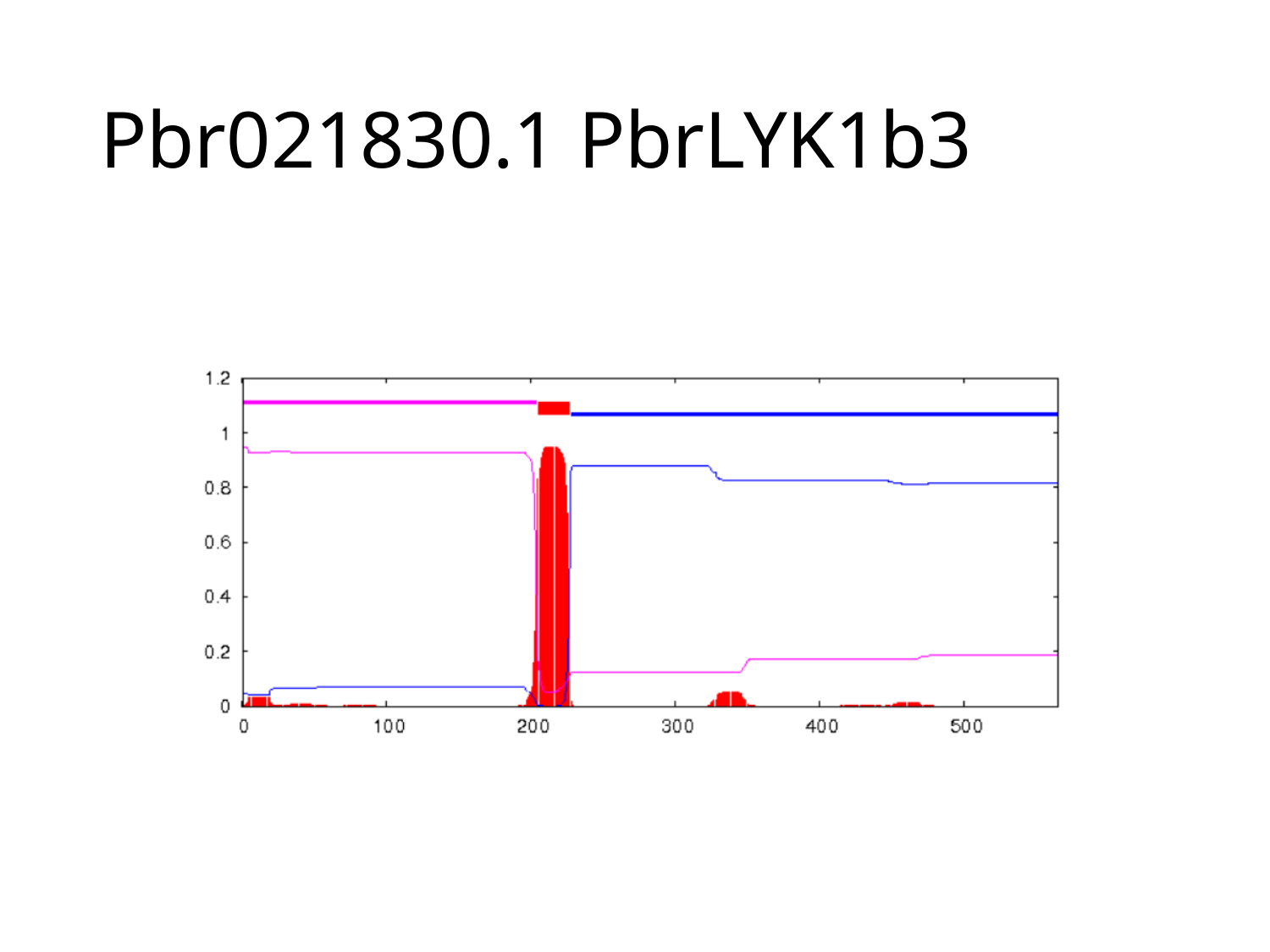

# Pbr021830.1 PbrLYK1b3

## Slide 6
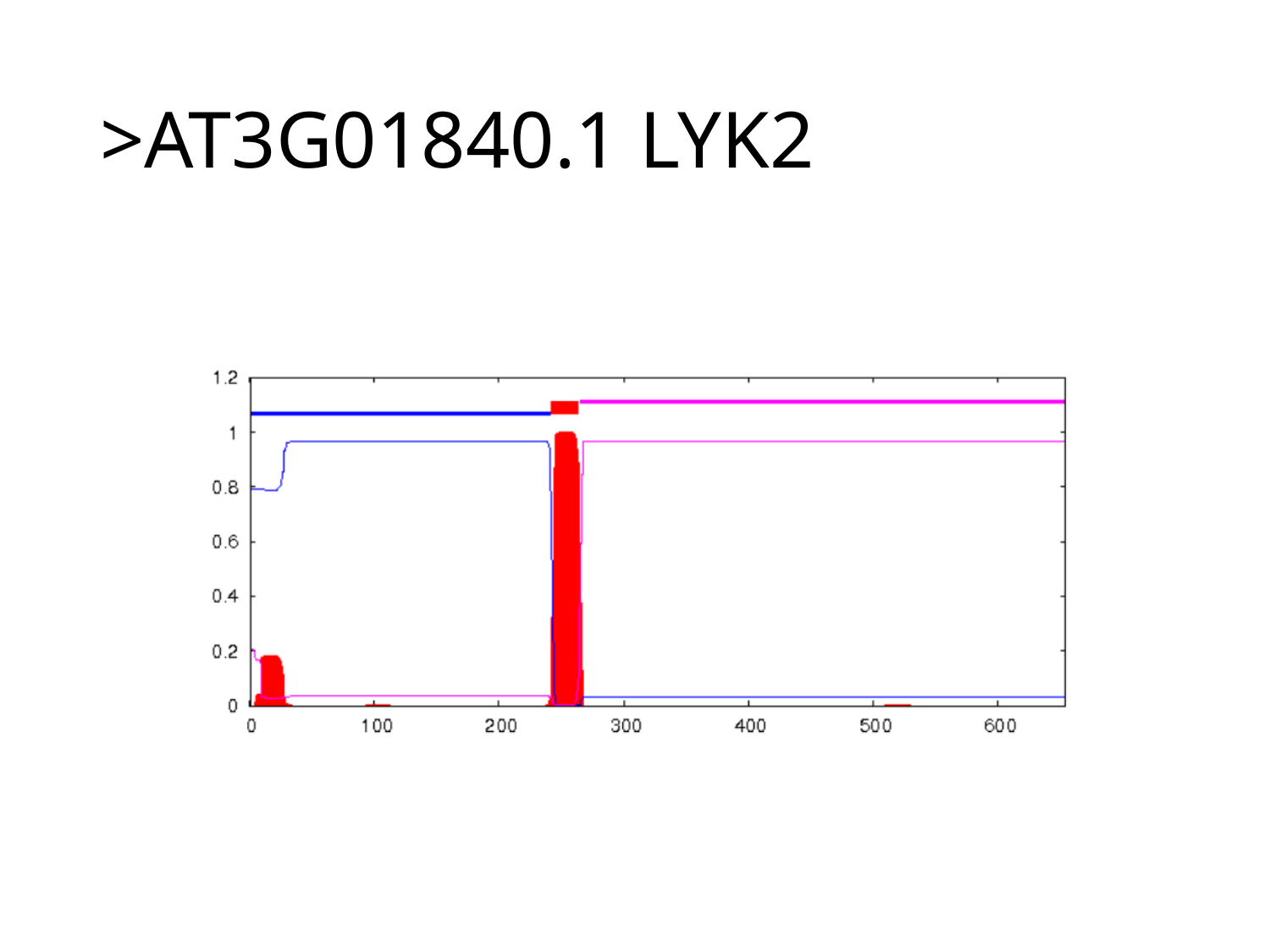

# >AT3G01840.1 LYK2

## Slide 7
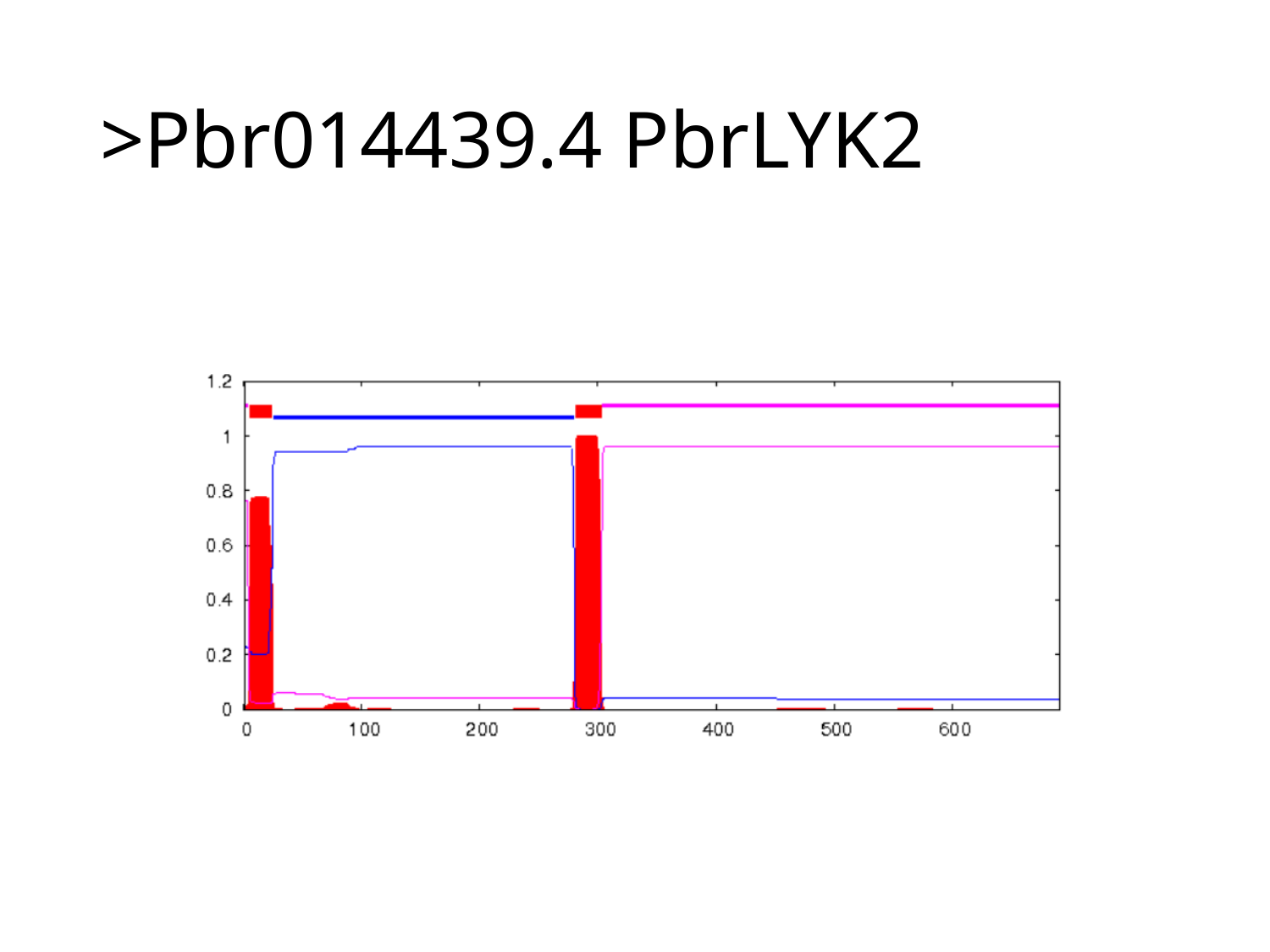

# >Pbr014439.4 PbrLYK2

## Slide 8
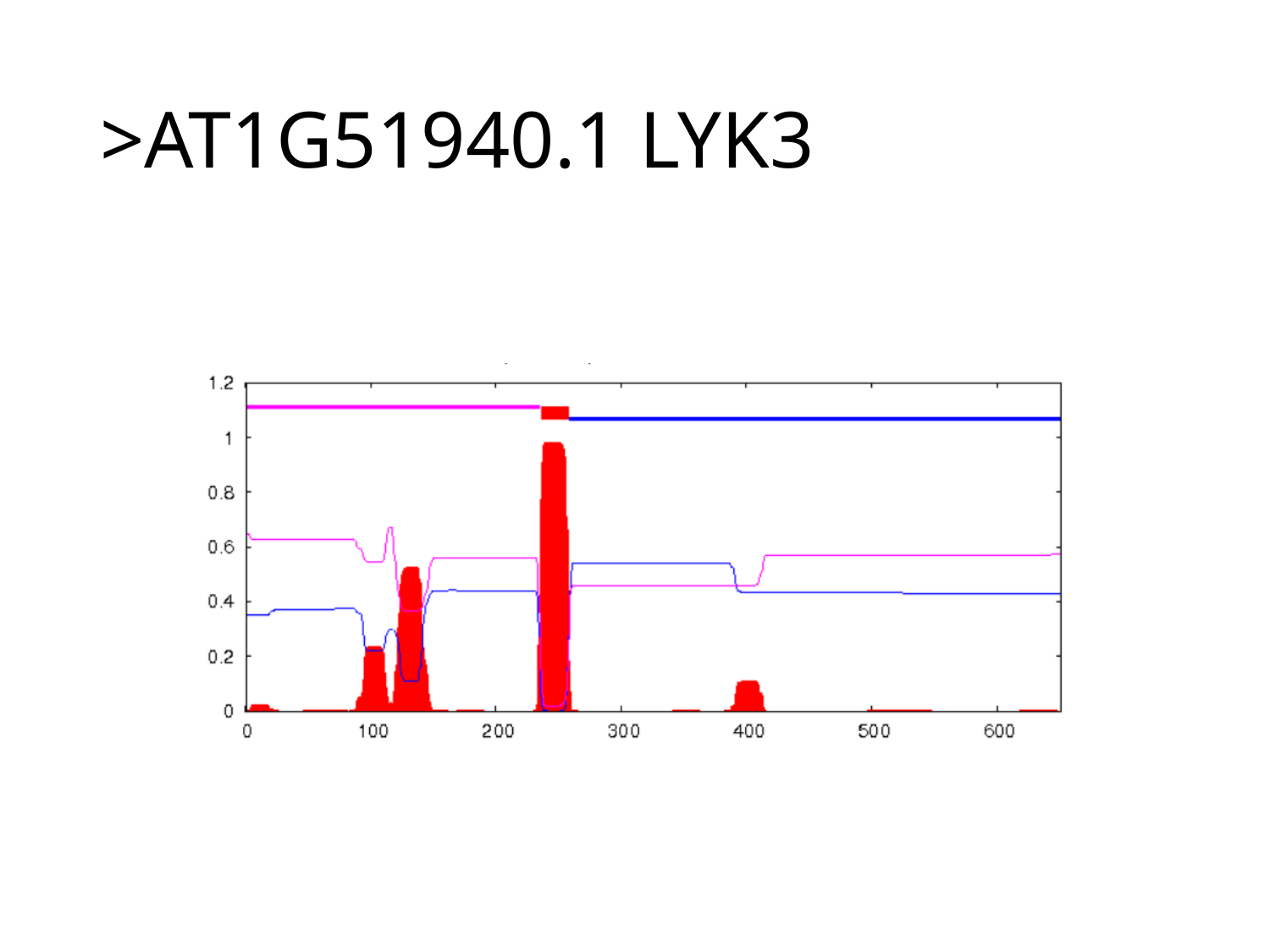

# >AT1G51940.1 LYK3

## Slide 9
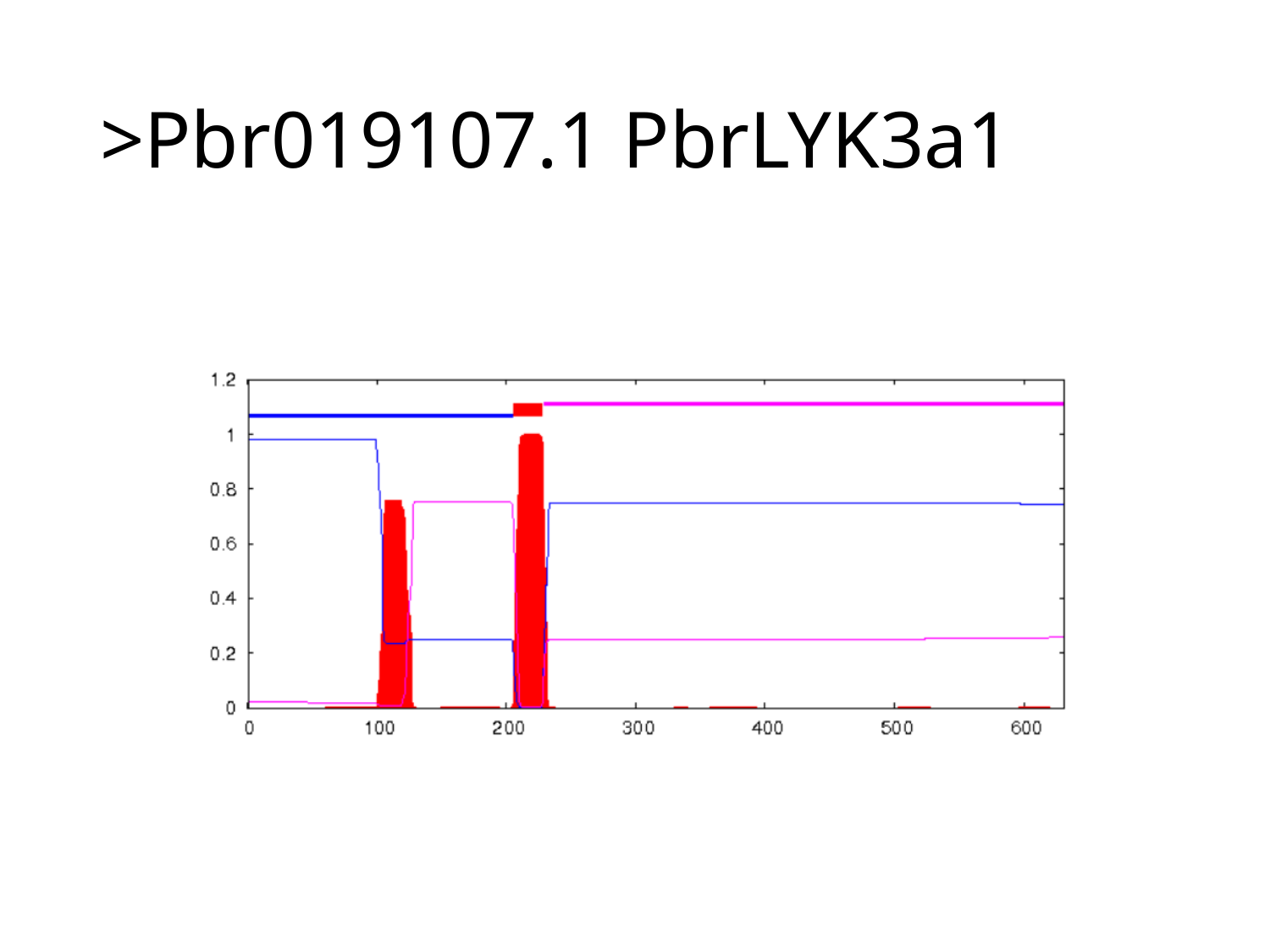

# >Pbr019107.1 PbrLYK3a1

## Slide 10
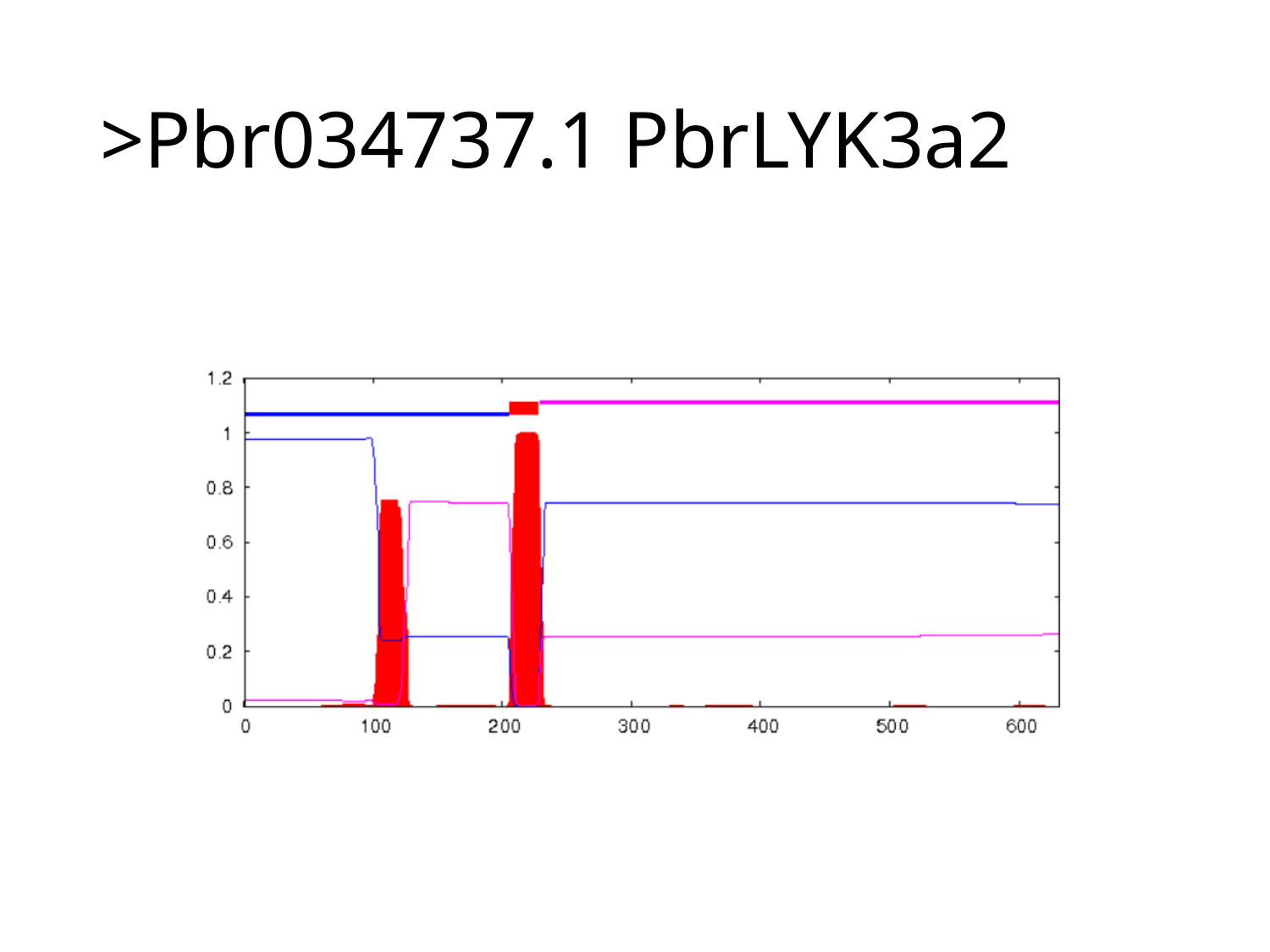

# >Pbr034737.1 PbrLYK3a2

## Slide 11
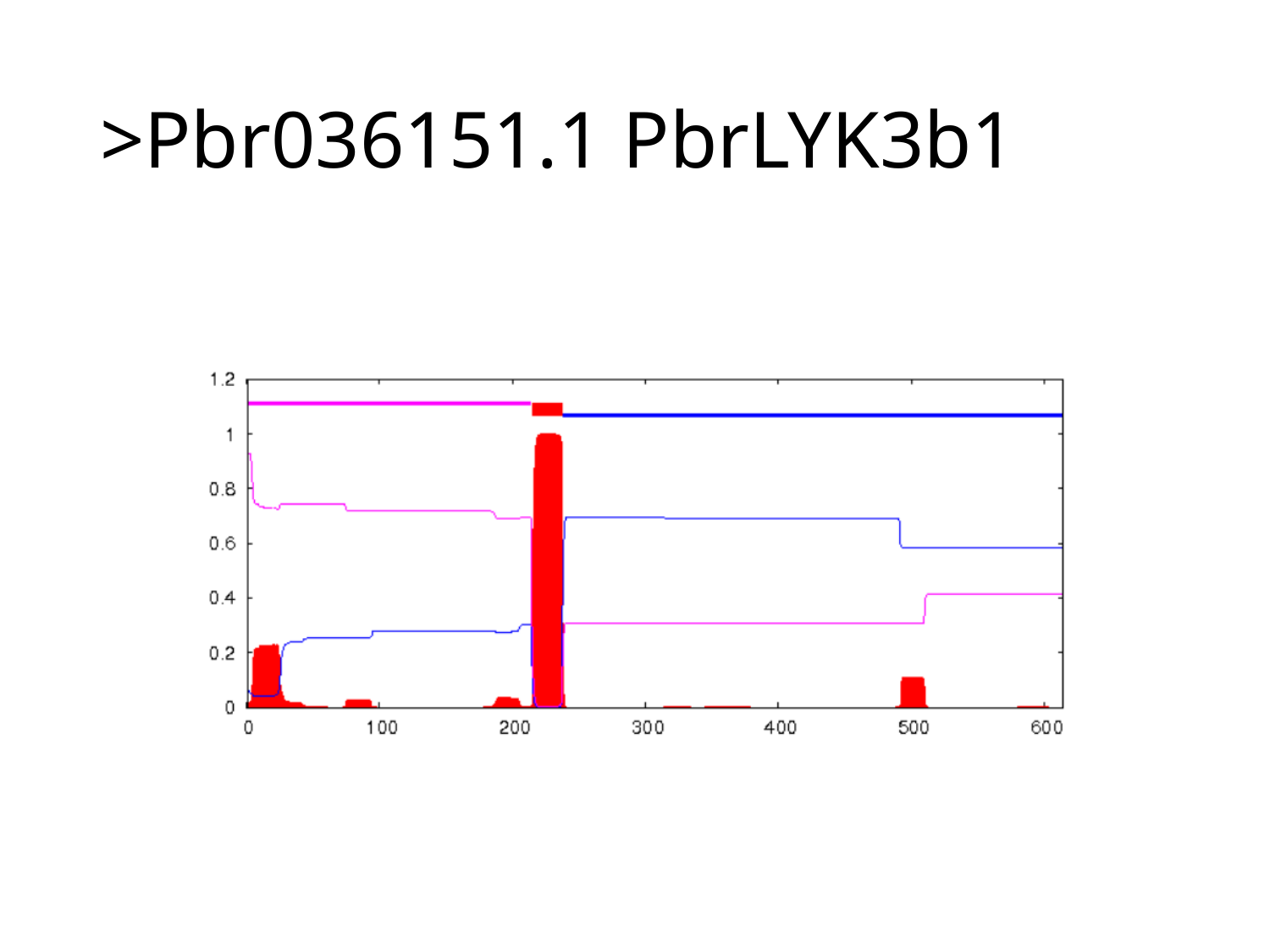

# >Pbr036151.1 PbrLYK3b1

## Slide 12
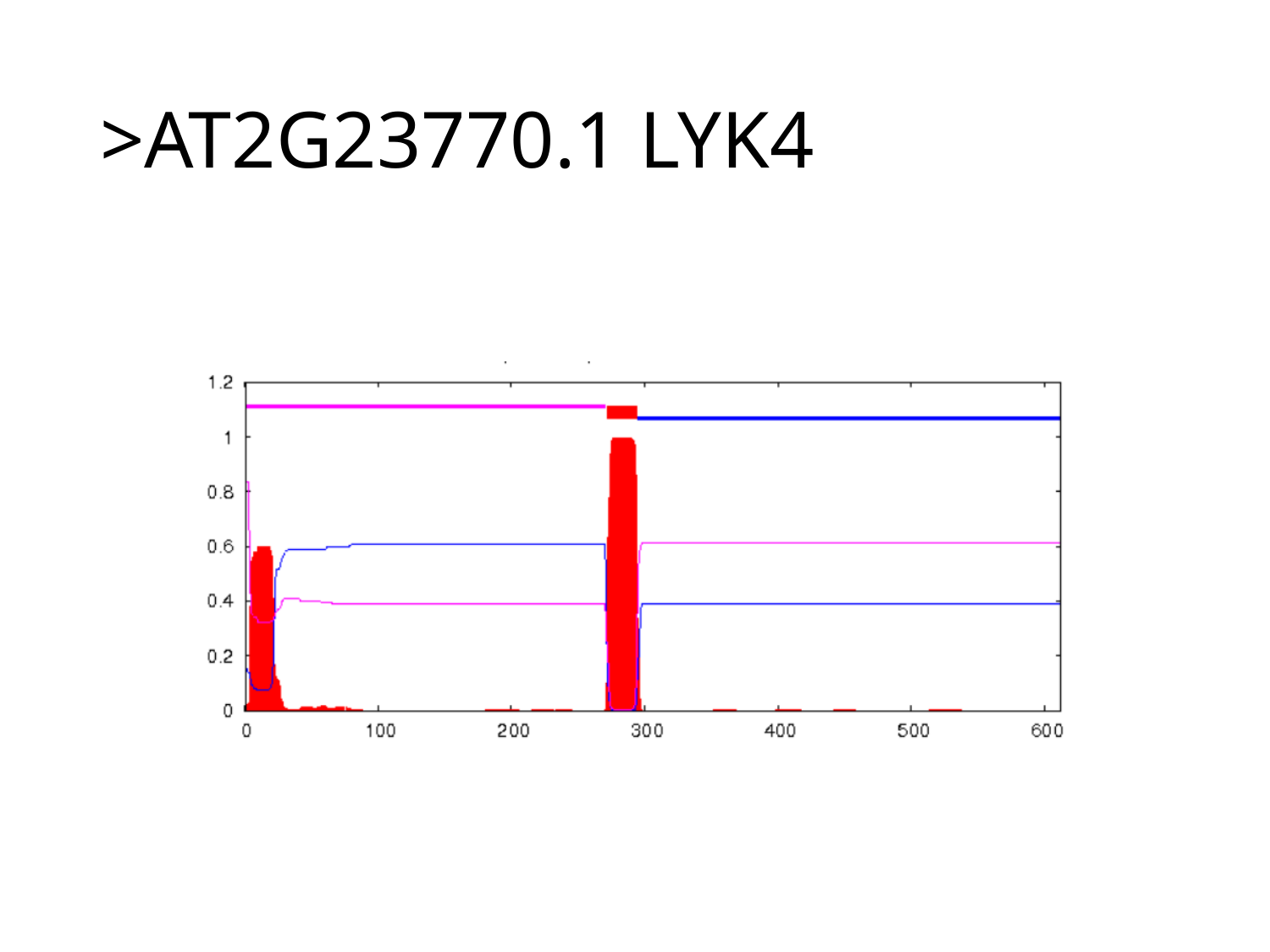

# >AT2G23770.1 LYK4

## Slide 13
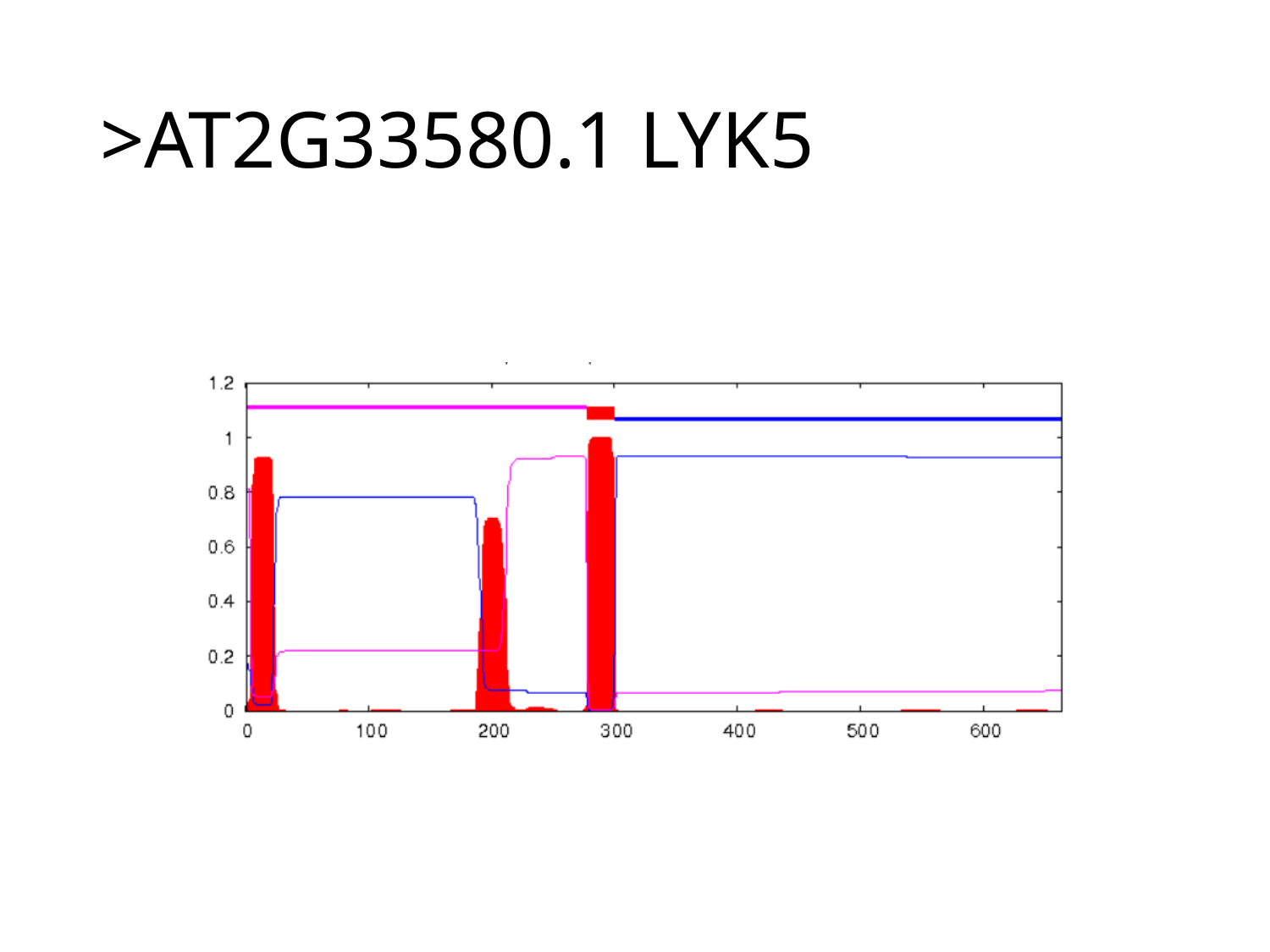

# >AT2G33580.1 LYK5

## Slide 14
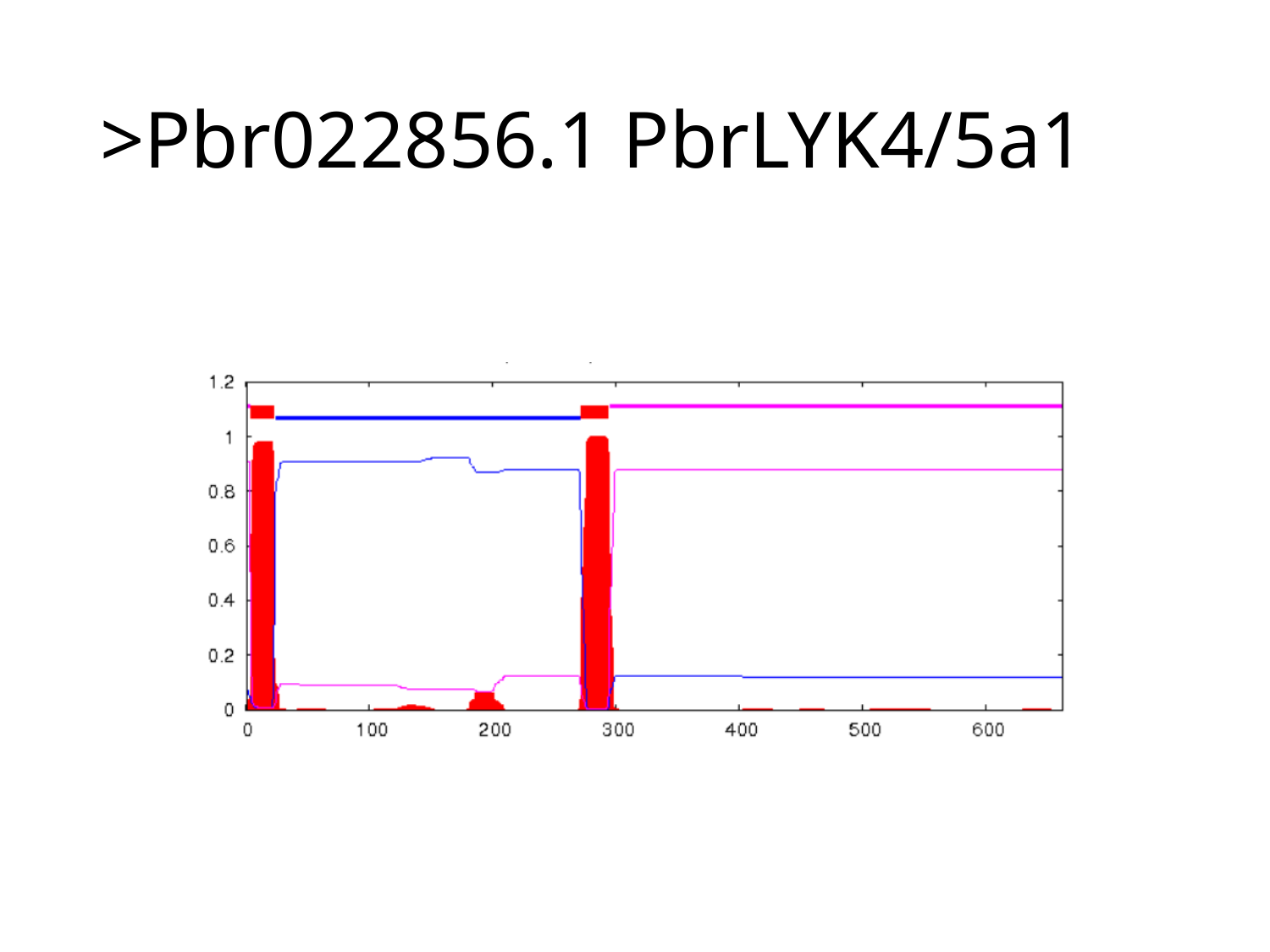

# >Pbr022856.1 PbrLYK4/5a1

## Slide 15
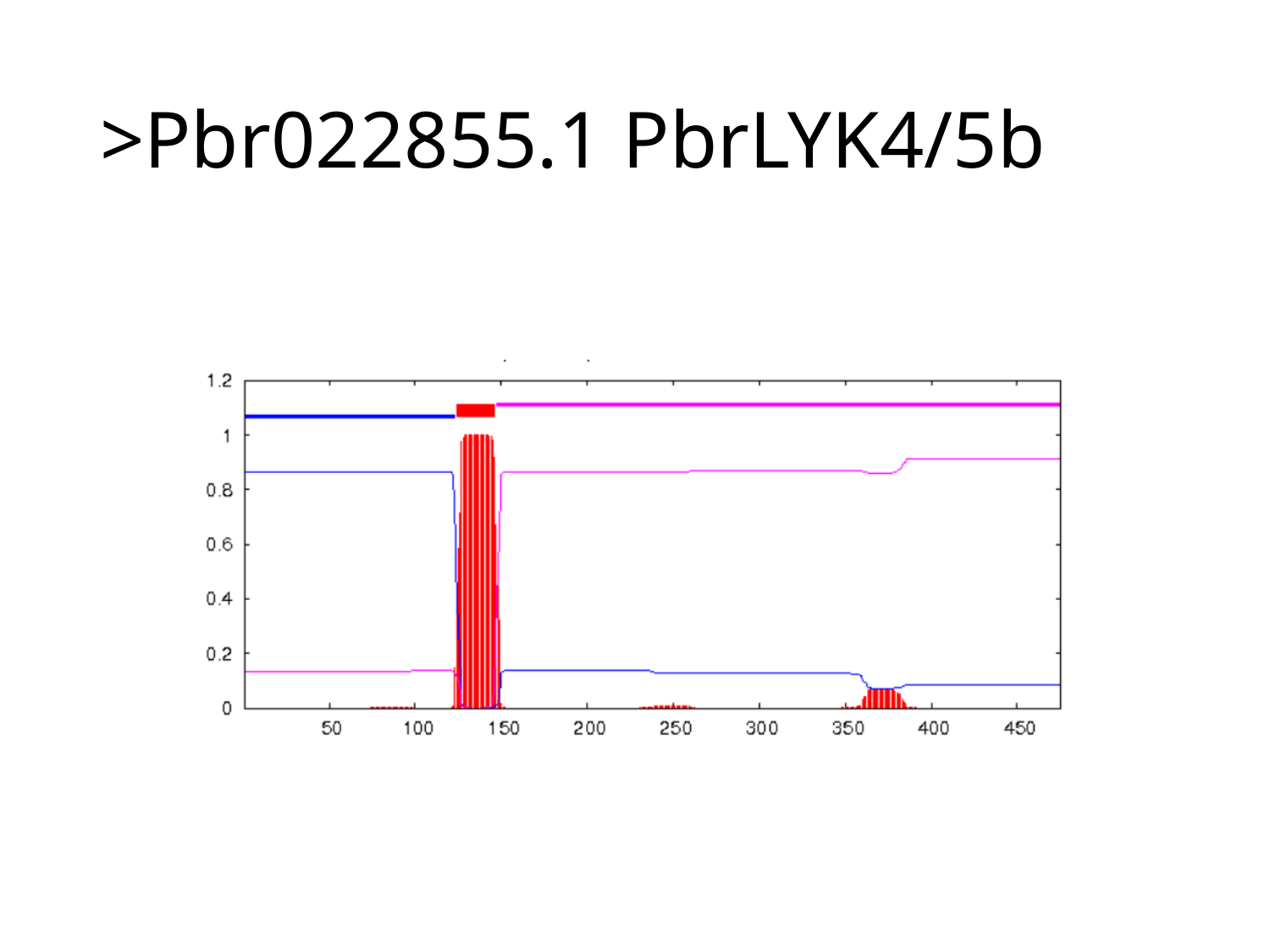

# >Pbr022855.1 PbrLYK4/5b

## Slide 16
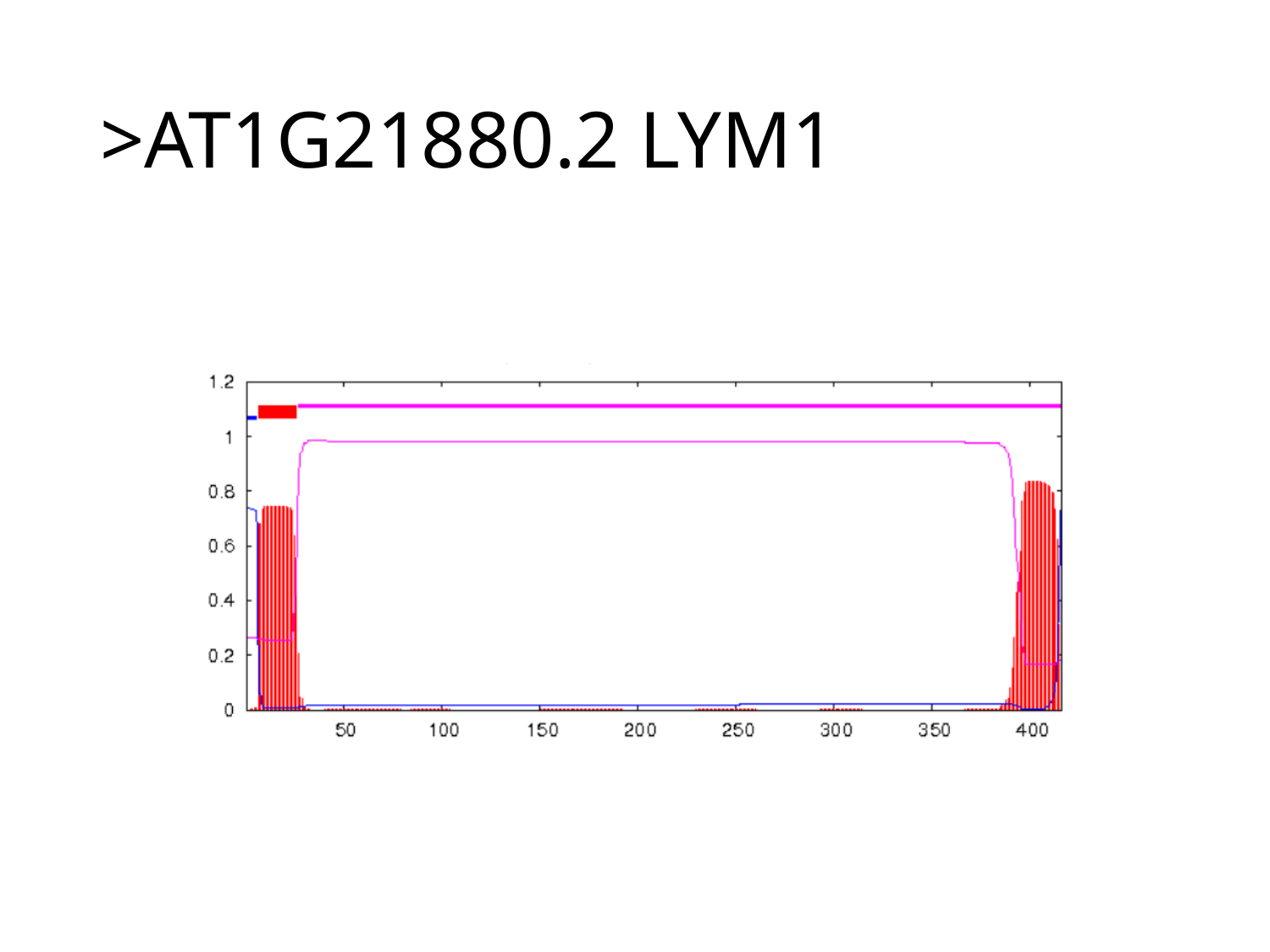

# >AT1G21880.2 LYM1

## Slide 17
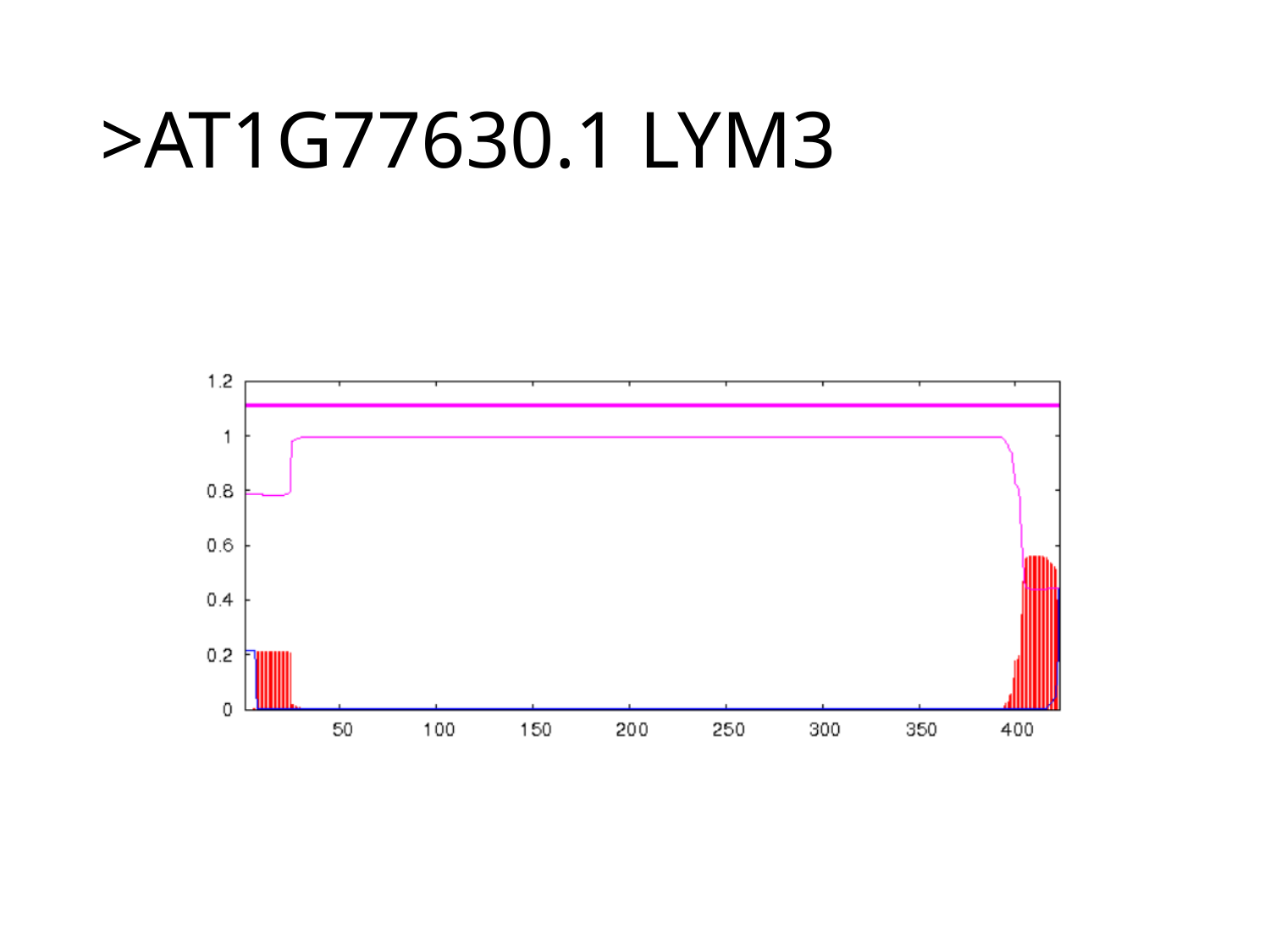

# >AT1G77630.1 LYM3

## Slide 18
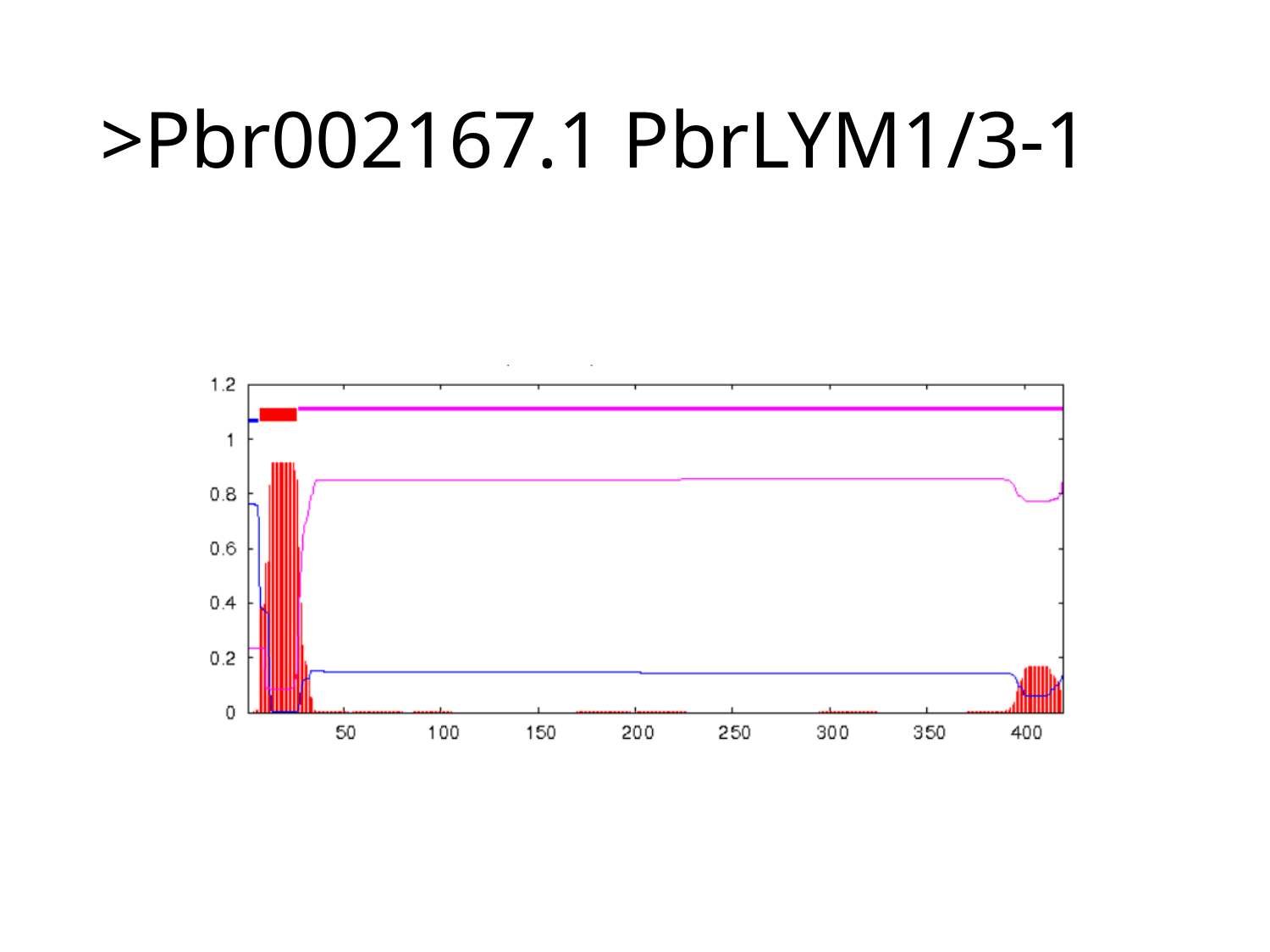

# >Pbr002167.1 PbrLYM1/3-1

## Slide 19
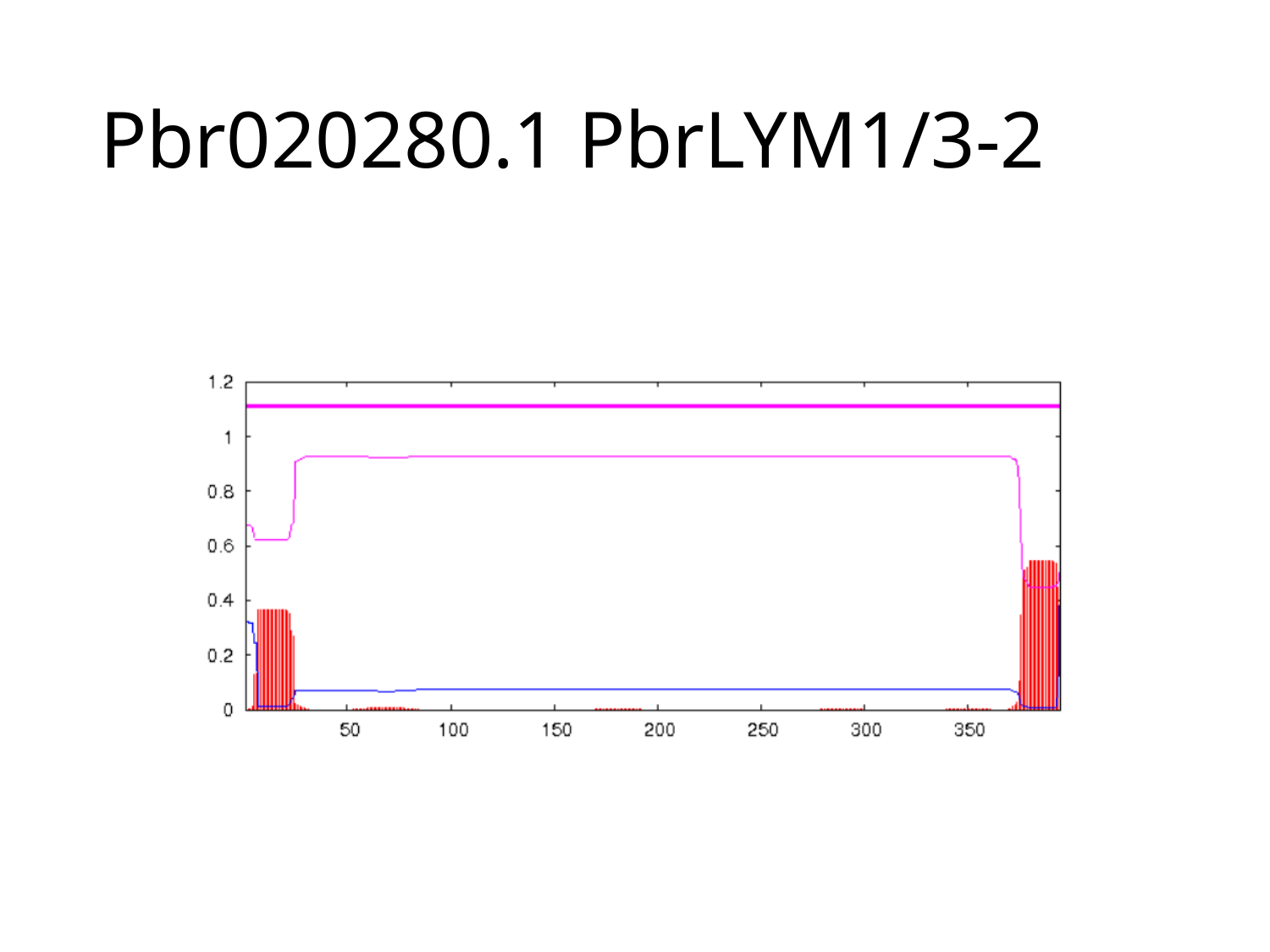

# Pbr020280.1 PbrLYM1/3-2

## Slide 20
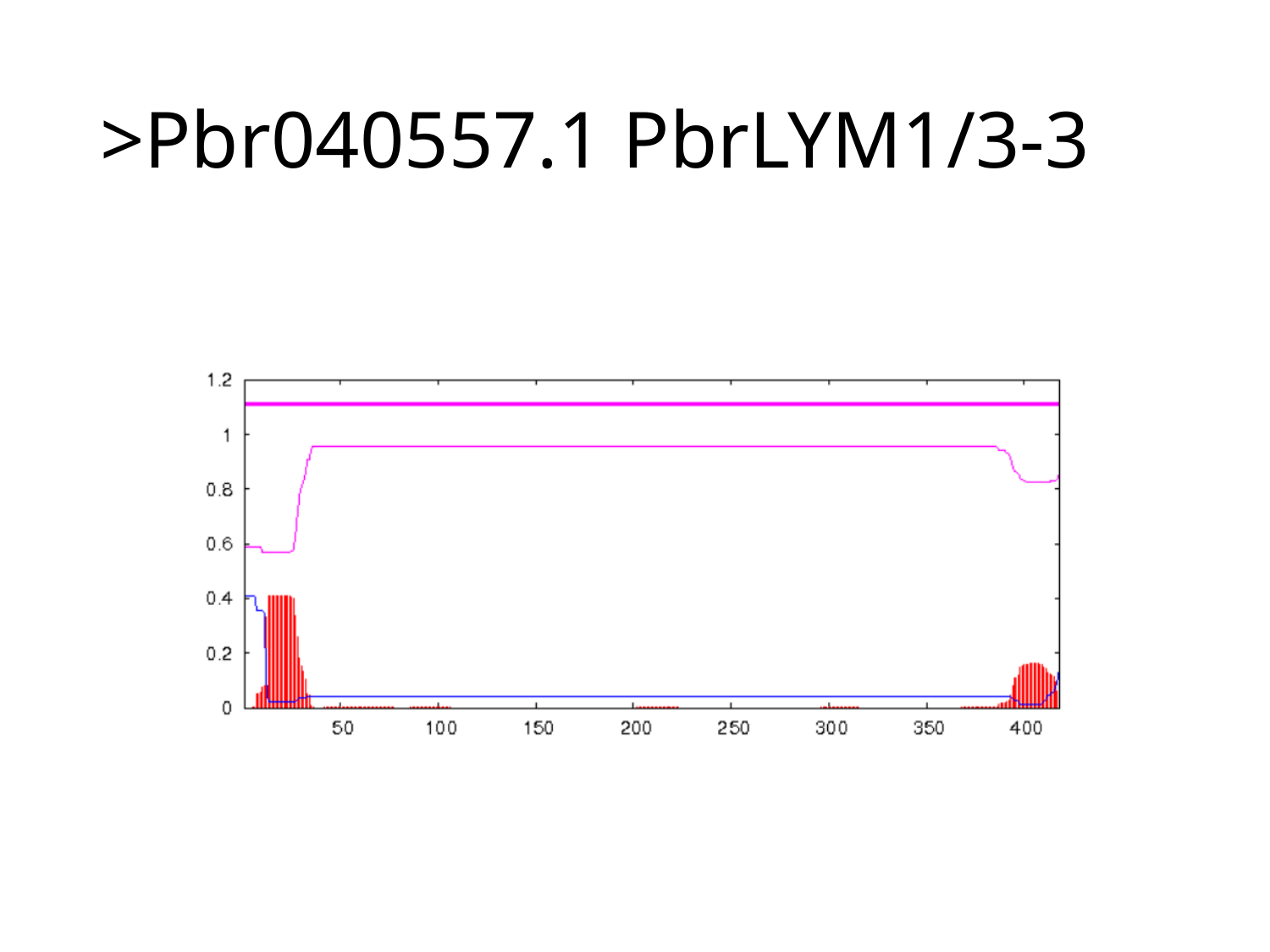

# >Pbr040557.1 PbrLYM1/3-3

## Slide 21
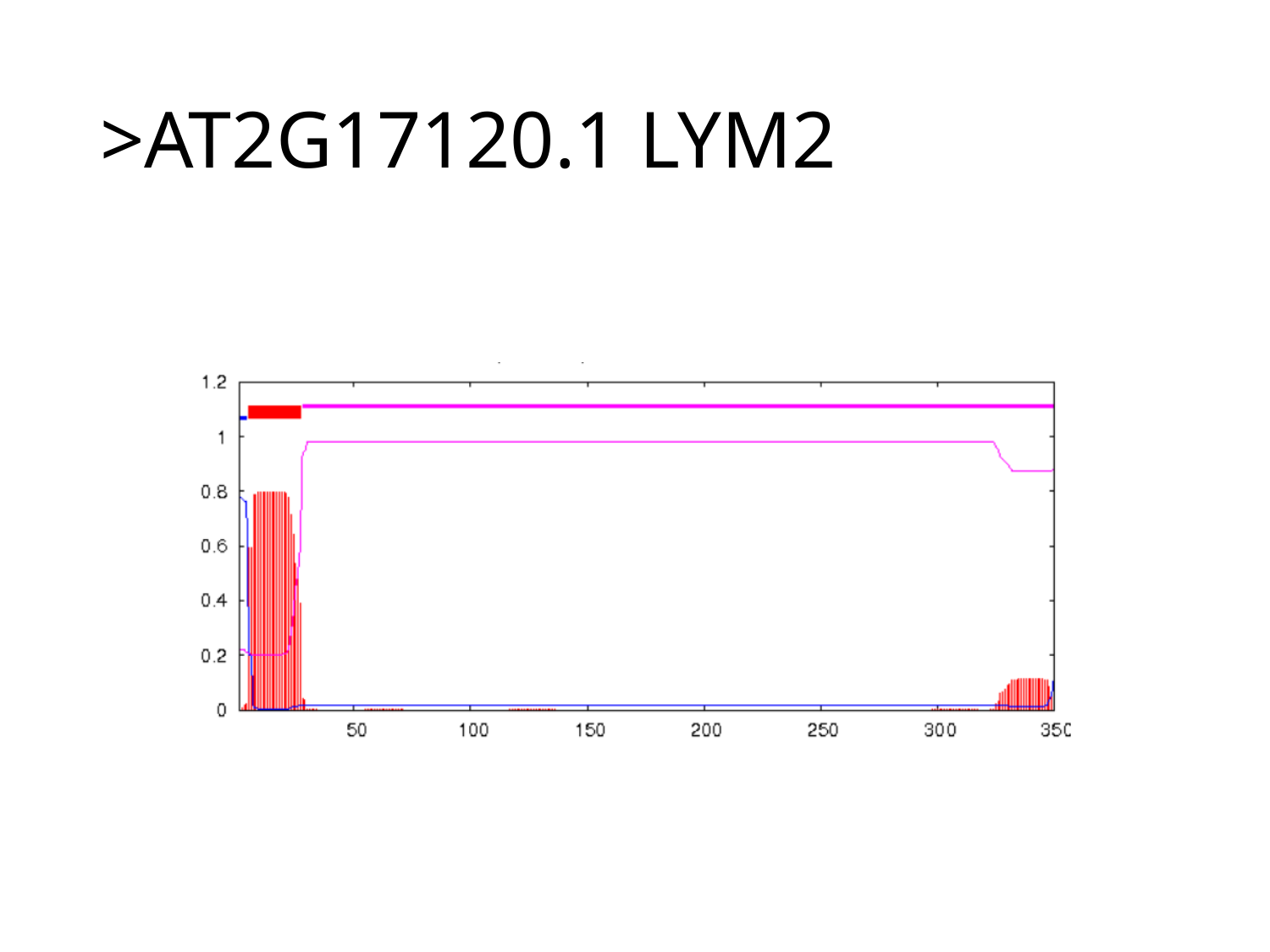

# >AT2G17120.1 LYM2

## Slide 22
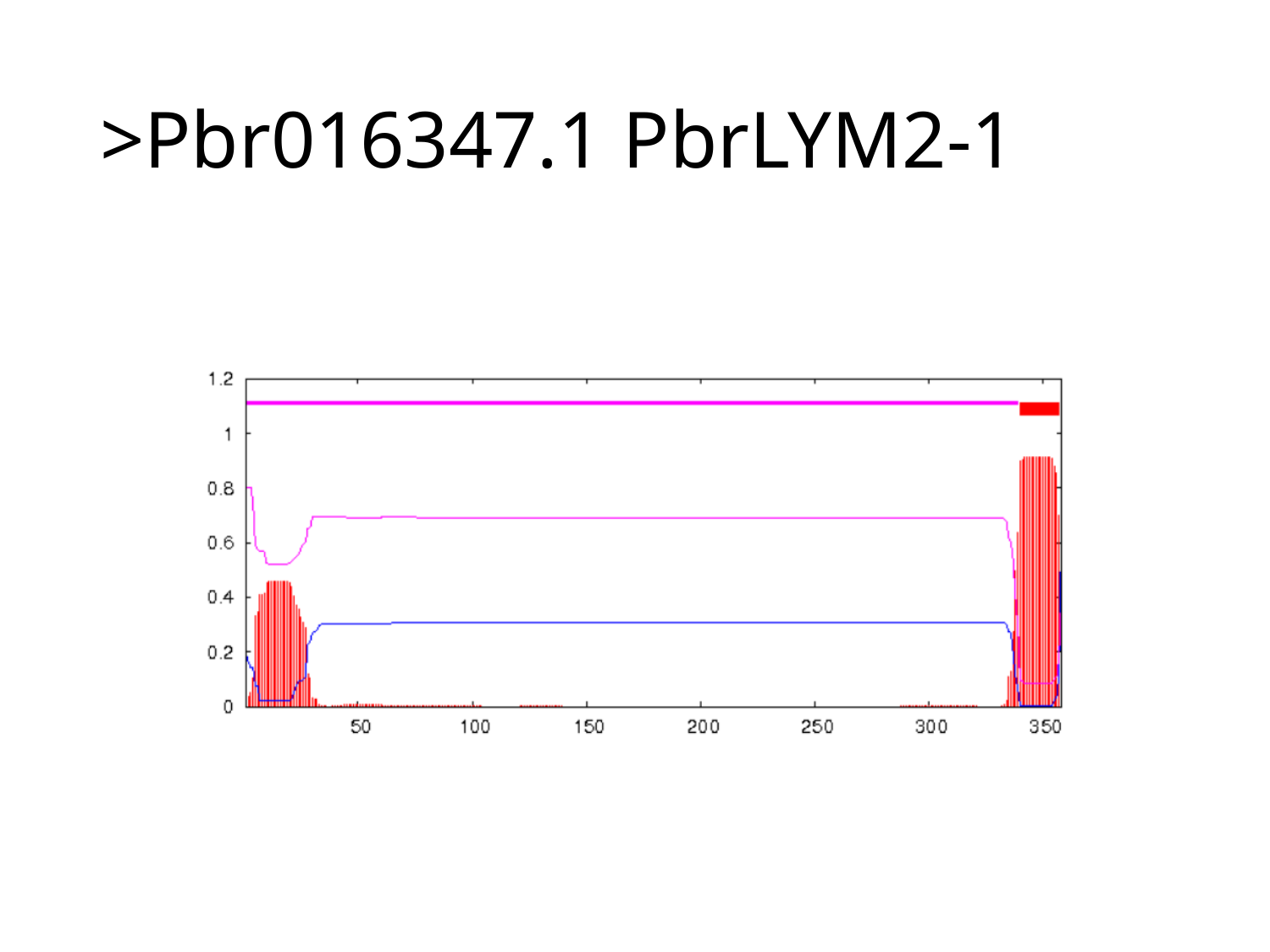

# >Pbr016347.1 PbrLYM2-1

## Slide 23
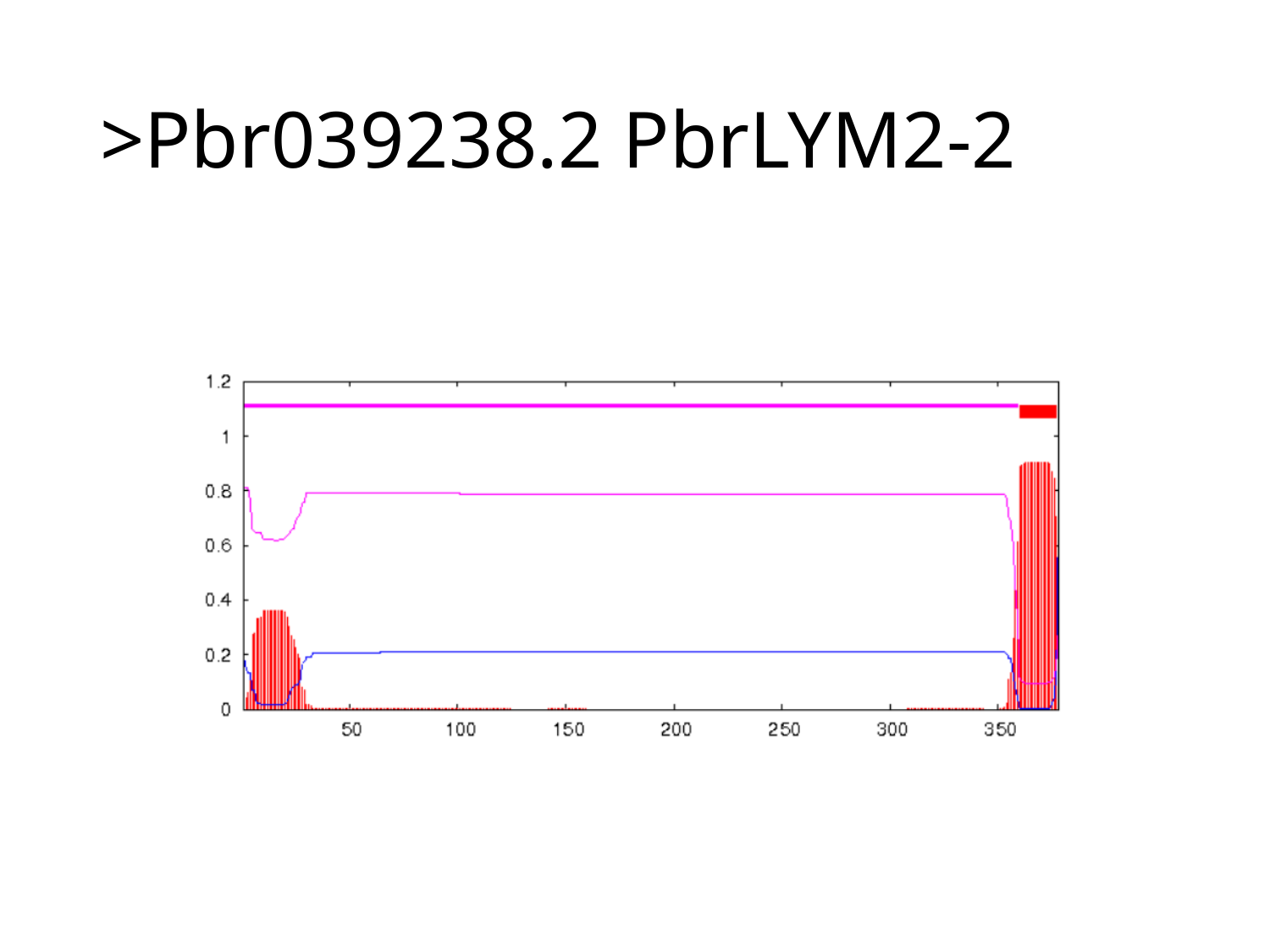

# >Pbr039238.2 PbrLYM2-2

## Slide 24
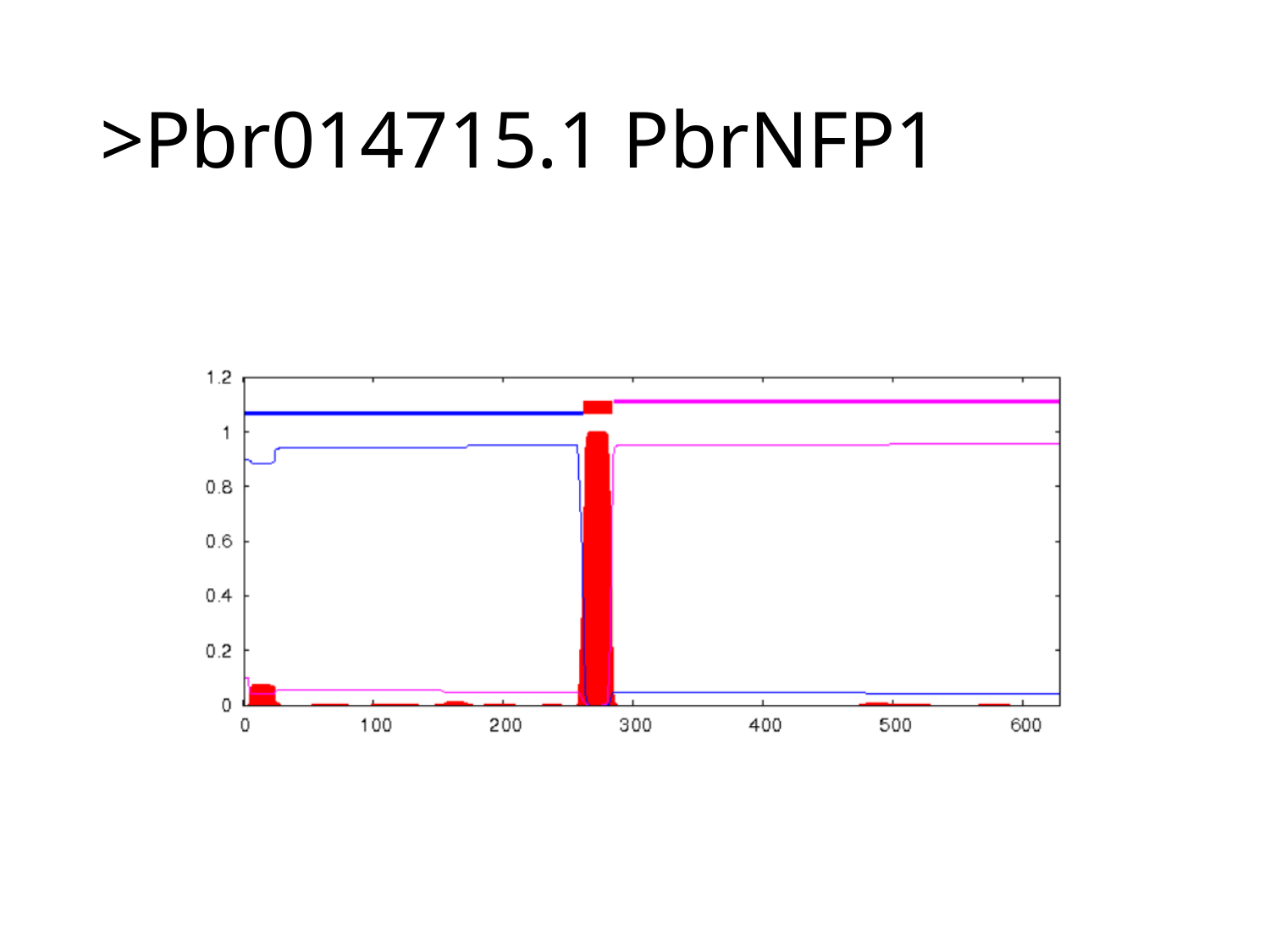

# >Pbr014715.1 PbrNFP1

## Slide 25
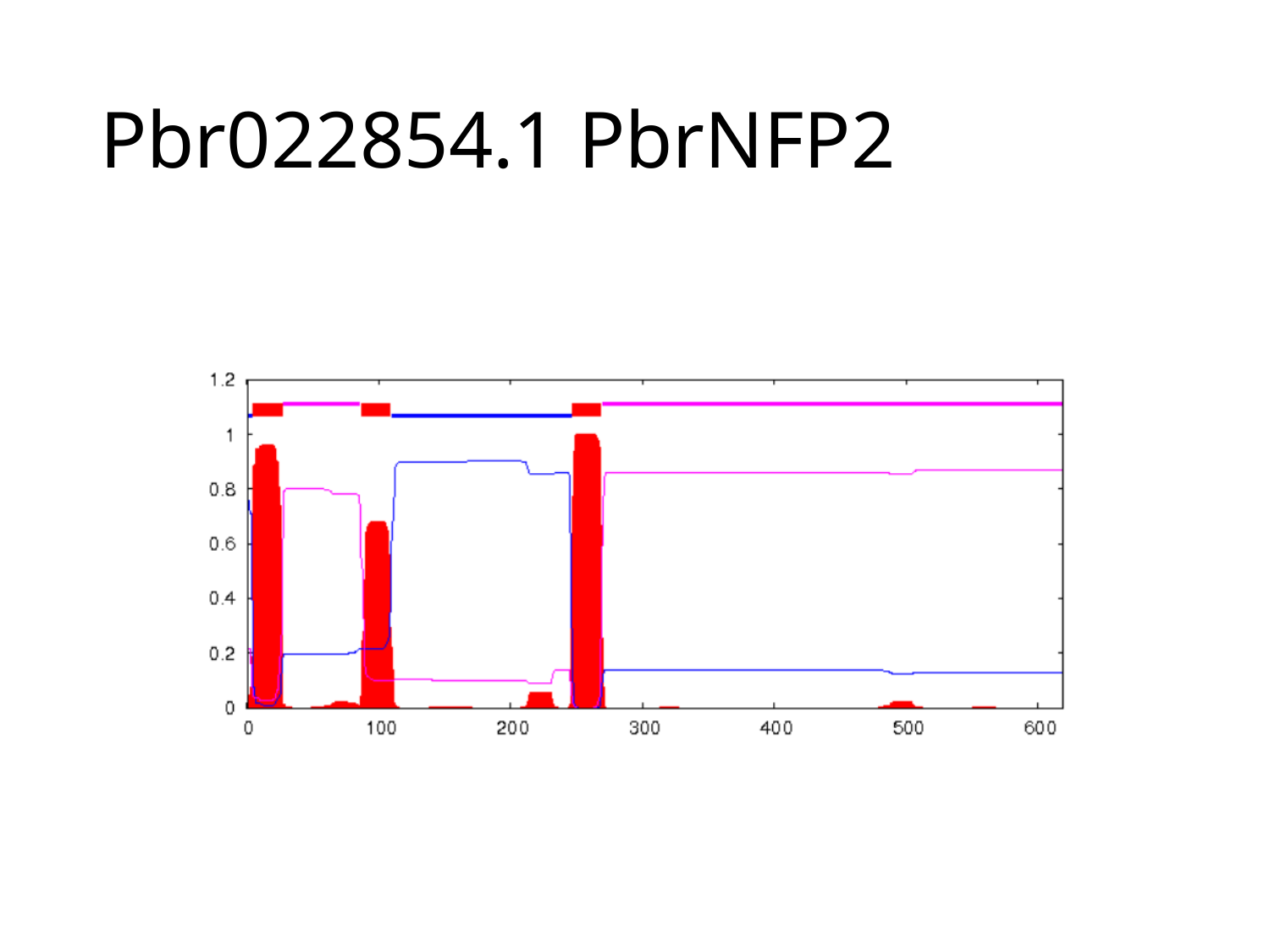

# Pbr022854.1 PbrNFP2

## Slide 26
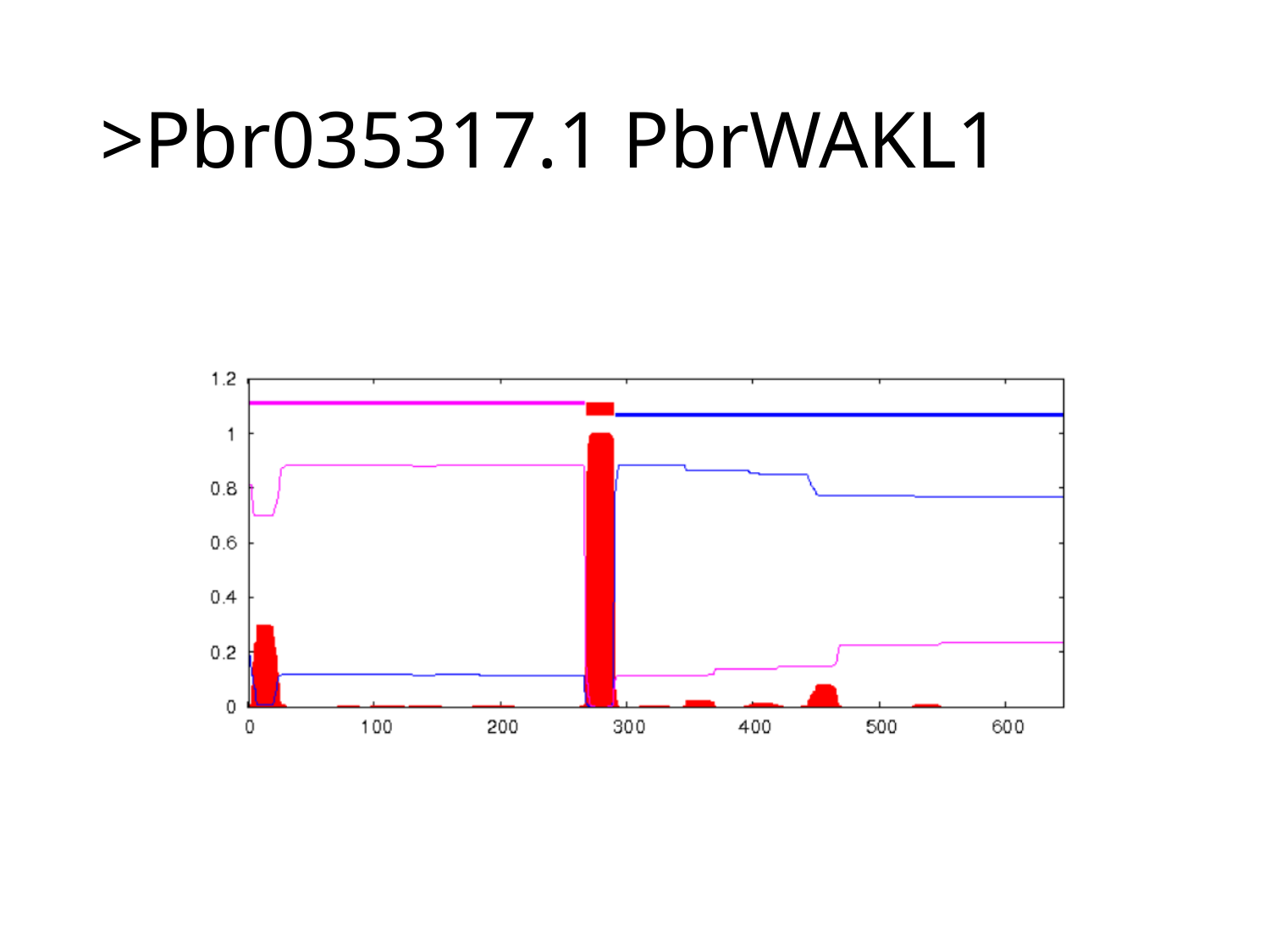

# >Pbr035317.1 PbrWAKL1
